# Mechanisms of Baicalin Alleviates Intestinal Inflammation: Role of M1 Macrophage Polarization and *Lactobacillus amylovorus*

**DOI:** 10.1002/advs.202415948

**Published:** 2025-04-08

**Authors:** Shunfen Zhang, Ruqing Zhong, Miao Zhou, Kai Li, Huiyuan Lv, Huixin Wang, Ye Xu, Dadan Liu, Qiugang Ma, Liang Chen, Hongfu Zhang

**Affiliations:** ^1^ State Key Laboratory of Animal Nutrition and Feeding, Key Laboratory of Animal Nutrition and Feed Science of Ministry of Agriculture and Rural Affairs, Institute of Animal Science Chinese Academy of Agricultural Sciences Beijing 100193 China; ^2^ College of Animal Science and Technology China Agricultural University Beijing 100193 China; ^3^ College of Animal Science and Technology Hunan Agricultural University Changsha 410128 China

**Keywords:** baicalin, *E. coli*, intestinal inflammation, *Lactobacillus amylovorus*, macrophages polarization, TLR4

## Abstract

Baicalin has been widely used for its anti‐inflammatory pharmacological properties, yet its effects on bacterial intestinal inflammation and the mechanisms remain unclear. This study revealed that baicalin alleviates bacterial intestinal inflammation through regulating macrophage polarization and increasing *Lactobacillus amylovorus* abundance in colon. Specifically, transcriptomic analysis showed that baicalin restored *Escherichia coli*‐induced genes expression changes including T helper cell 17 differentiation‐related genes, macrophage polarization related genes, and TLR/IRF/STAT signaling pathway. Subsequent microbial and non‐targeted metabolomic analysis revealed that these changes may be related to the enhancement of *Lactobacillus amylovorus* and the upregulation of its metabolites including chrysin, lactic acid, and indoles. Furthermore, whole‐genome sequencing of *Lactobacillus amylovorus* provided insights into its functional potential and metabolic annotations. *Lactobacillus amylovorus* supplementation alleviates *Escherichia coli*‐induced intestinal inflammation in mice and similarly inhibited M1 macrophage polarization through TLR4/IRF/STAT pathway. Additionally, baicalin, *Lactobacillus amylovorus*, or chrysin alone could regulate macrophage polarization, highlighting their independent anti‐inflammatory potential. Notably, this study revealed that baicalin alleviates intestinal inflammation through TLR4/IRF/STAT pathway and increasing *Lactobacillus amylovorus* abundance and the synthesis of chrysin. These findings provide new insights into the therapeutic potential of baicalin and *Lactobacillus amylovorus* in preventing and treating intestinal inflammation, offering key targets for future interventions.

## Graphical abstract

Baicalin alleviates *E. coli*‐induced intestinal inflammation through dual mechanisms: direct inhibition of the TLR4/IRF/STAT signaling pathway to modulate macrophage polarization and antigen presentation, thereby regulating Th17/Treg cell differentiation; and microbiota‐mediated indirect effects via increasing *Lactobacillus amylovorus* abundance and chrysin production. Notably, *Lactobacillus amylovorus* exerts anti‐inflammatory activity through TLR4/IRF/STAT pathway and appears to be a key bacterial strain in baicalin metabolism.

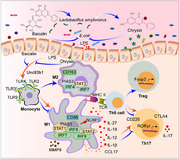

## 1 Introduction

Bacterial intestinal inflammation is a globally prevalent health issue, posing a significant threat to public health. The World Health Organization statistics indicate that ≈525 million people worldwide are infected with bacterial intestinal pathogens each year, of which 220 million are infected with pathogenic *Escherichia coli* (*E. coli*), resulting in ≈100 000 deaths.^[^ ^1^, ^2^ ^]^ Children under five are the primary affected group. Macrophages are primary antigen‐presenting cells that express toll‐like receptors (TLRs) on their surface after phagocytosis of foreign substances and present antigens through major histocompatibility (MHC) II molecules to activate T cells.^[^ ^3^ ^]^ The T cell receptor (TCR) on T cells interacts with the MHC‐antigen complex and triggers downstream signaling to differentiate naive T cells into specific subtypes.^[^ ^3^ ^]^ Naive macrophages (M0) can polarize into the pro‐inflammatory M1 phenotype or the pro‐regenerative M2 phenotype.^[^ ^4^ ^]^ Persistent M1 macrophage activation can result in tissue damage, making regulating the M1/M2 balance crucial for preventing excessive inflammation.^[^ ^5^ ^]^ Th17 cells are a subset of CD4^+^ T cells that secrete interleukin‐17 (IL‐17) and are primarily involved in pro‐inflammatory responses and defense against extracellular pathogens.^[^ ^6^ ^]^ Retinoic acid‐related orphan receptor γt (RORγt) is a key transcription factor for their differentiation. In contrast, Treg cells are a subset of CD4^+^ T cells with immunosuppressive functions, inhibiting other immune cells' activity by secreting inhibitory cytokines (IL‐10 and TGF‐β).^[^ ^7^ ^]^ Forkhead box protein P3 (FOXP3) is the core transcription factor for Treg cells' differentiation and functional maintenance. The differentiation and functions of Th17 and Treg cells are mutually antagonistic, and an imbalance in their ratio is associated with various diseases, including inflammatory and autoimmune disorders.^[^ ^8^ ^]^ Our previous study has demonstrated that maintaining the Th17/Treg balance is a key target for treating bacterial‐induced intestinal inflammation.^[^ ^9^ ^]^

Currently, the primary treatment for *E. coli*‐induced intestinal inflammation is antibiotic therapy. However, the misuse of antibiotics has resulted in the emergence of resistant strains, significantly reducing treatment efficacy.^[^ ^10^ ^]^ This has sparked interest in alternative therapeutic strategies, including those based on natural products. *Scutellaria baicalensis Georgi*, a traditional Chinese herb, is extensively used to treat various conditions, including malaria, enteritis, and diarrhea.^[^ ^11^ ^]^ Its primary active ingredient, baicalin, is a flavonoid with various pharmacological properties, including anti‐inflammatory, antioxidant, and antimicrobial effects.^[^ ^12^, ^13^, ^14^ ^]^ Multiple studies on baicalin have focused on its ability to alleviate ulcerative colitis through immune‐modulatory pathways, including reducing inflammatory cytokines, inhibiting oxidative stress, and modulating macrophage migration factors.^[^ ^12^, ^15^, ^16^, ^17^ ^]^ However, baicalin's bioavailability is low, at only 2.2%, and its absorption depends on gut microbiota to metabolize it into baicalein.^[^ ^18^ ^]^ Specifically, baicalin is hydrolyzed by gut bacterial *β*‐glucuronidase into baicalein, which is then absorbed and reconverted into baicalin in the intestine through UDP‐glucuronosyltransferase activity.^[^ ^19^, ^20^ ^]^ Gut microbiota is essential for the absorption and metabolism of traditional herbal medicines, as well as for the modulation of immune responses and the promotion of overall health.^[^ ^21^ ^]^ Although studies have shown that baicalin can regulate microbial composition,^[^ ^13^, ^22^ ^]^ the specific mechanisms underlying its impact and the contribution of microbes to its anti‐inflammatory effects remain unclear. *Lactobacillus amylovorus* is a potential probiotic that can strengthen the intestinal barrier and promote lactose and carbohydrate utilization.^[^ ^23^ ^]^ However, its effects on the alleviation of intestinal inflammation remains unclear.

In this study, *E. coli* was used to construct a bacterial intestinal inflammation model to explore the alleviating effect of baicalin. The results demonstrate for the first time that baicalin not only directly influences macrophage polarization and T cell differentiation but also increases *Lactobacillus amylovorus* abundance and the synthesis of chrysin to alleviate intestinal inflammation. These findings highlight the critical role of *Lactobacillus amylovorus* in the therapeutic effects of baicalin.

## 2 Results

### 2.1 Baicalin Alleviates *E. coli*‐Induced Intestinal Morphological Damage and Inflammation

**Figure 1:**
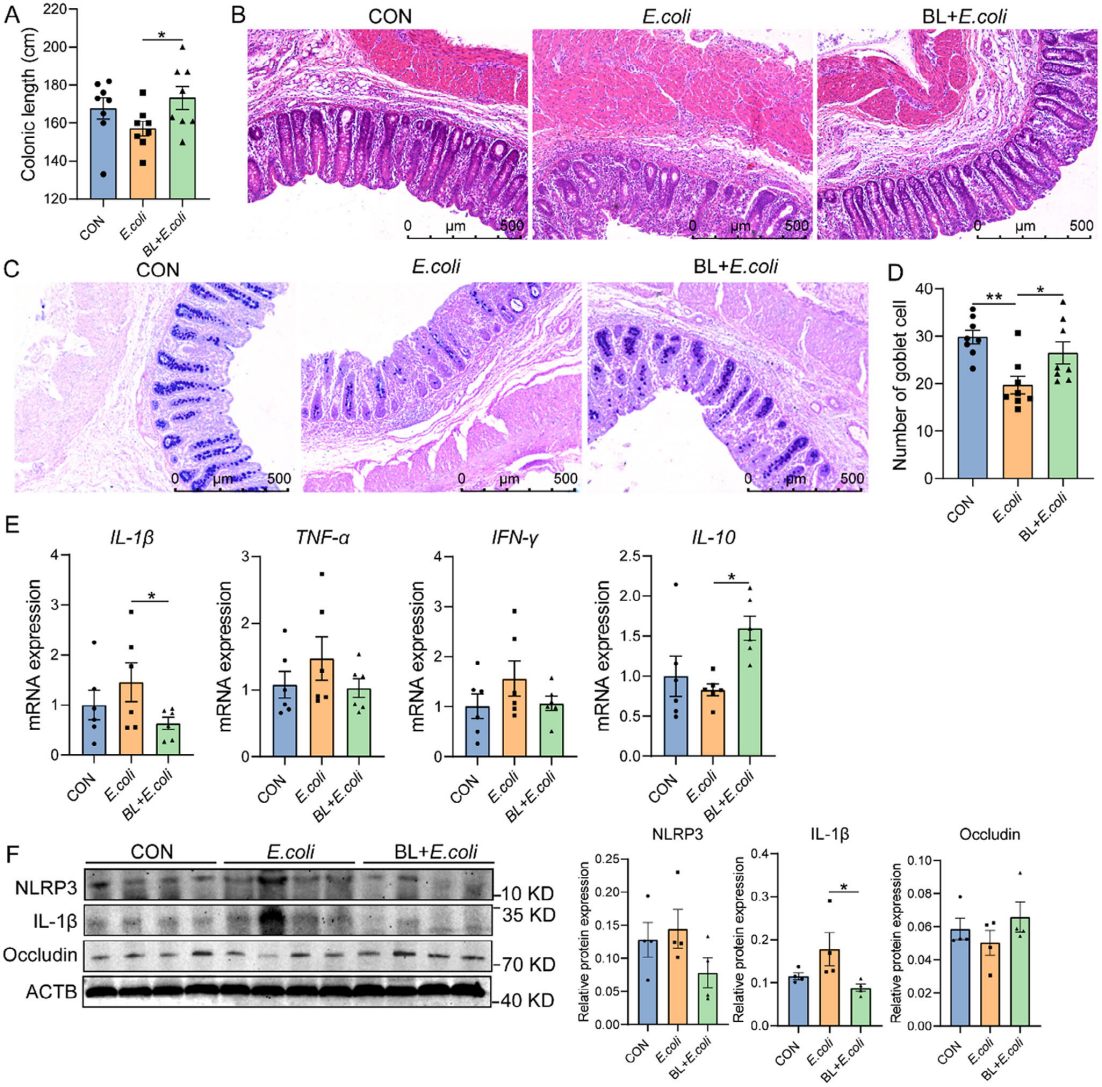
Baicalin alleviates *E. coli*‐induced colon damage and inflammation. A) Colonic length (*n* = 8). B) Hematoxylin and eosin (H&E) sections of colon. Scale bar: 500 µm. C) Representative AB‐PAS staining of colon tissue sections. Scale bar: 500 µm. D) Colonic goblet cell count (*n* = 8). E) mRNA expression of *IL‐1β*, *TNF‐α*, *IFN‐γ*, *IL‐10* (*n* = 6). F) Western blotting analyses of the expression of NLRP3, IL‐1β, occludin, and ACTB in the colon, and the ratio of cleaved to full‐length forms for these proteins was calculated (*n* = 4). ACTB was used as a loading control. Data are presented as mean ± SEM and statistical analysis was performed using one‐way ANOVA followed by the LSD test. ^*^ means *p*  <  0.05, ^**^ means *p* <  0.01.

To investigate the therapeutic effect of baicalin on bacterial intestinal inflammation, piglets were received a diet containing 100 mg kg^−1^ baicalin, and then the intestinal inflammation model was constructed by injecting of *E. coli*. The results showed that baicalin supplementation significantly increased colon length compared with the *E. coli* group (*P* < 0.05, **Figure** **1**A). The morphology of piglets in the control (CON) group was complete, and the crypts were arranged regularly. The crypts of piglets in the *E. coli* group were significantly damaged and disordered with some inflammatory infiltration (Figure 1B). However, the colonic morphology of piglets in the baicalin prevention (BL + *E. coli*) group was more complete, and the crypts were arranged more regularly than those in the *E. coli* group (Figure 1B). Alcian blue and periodic acid Schiff (AB‐PAS) staining revealed that baicalin significantly increased colonic goblet cells count (*P* < 0.05, Figure 1C,D). Additionally, baicalin supplementation significantly reduced the mRNA levels of the pro‐inflammatory cytokine *IL‐1β* and elevated the anti‐inflammatory cytokine *IL‐10* levels, compared to the *E. coli* group (*P* < 0.05, Figure 1E). Western blotting analysis revealed that IL‐1β expression in the BL + *E. coli* group was significantly lower than the *E. coli* group (*P* < 0.05, Figure 1F), and NLRP3 expression exhibited the same trend. These results indicate that baicalin can alleviate *E. coli*‐induced intestinal inflammation.

### 2.2 Baicalin Regulates Macrophage Polarization and T Cell Differentiation

**Figure 2:**
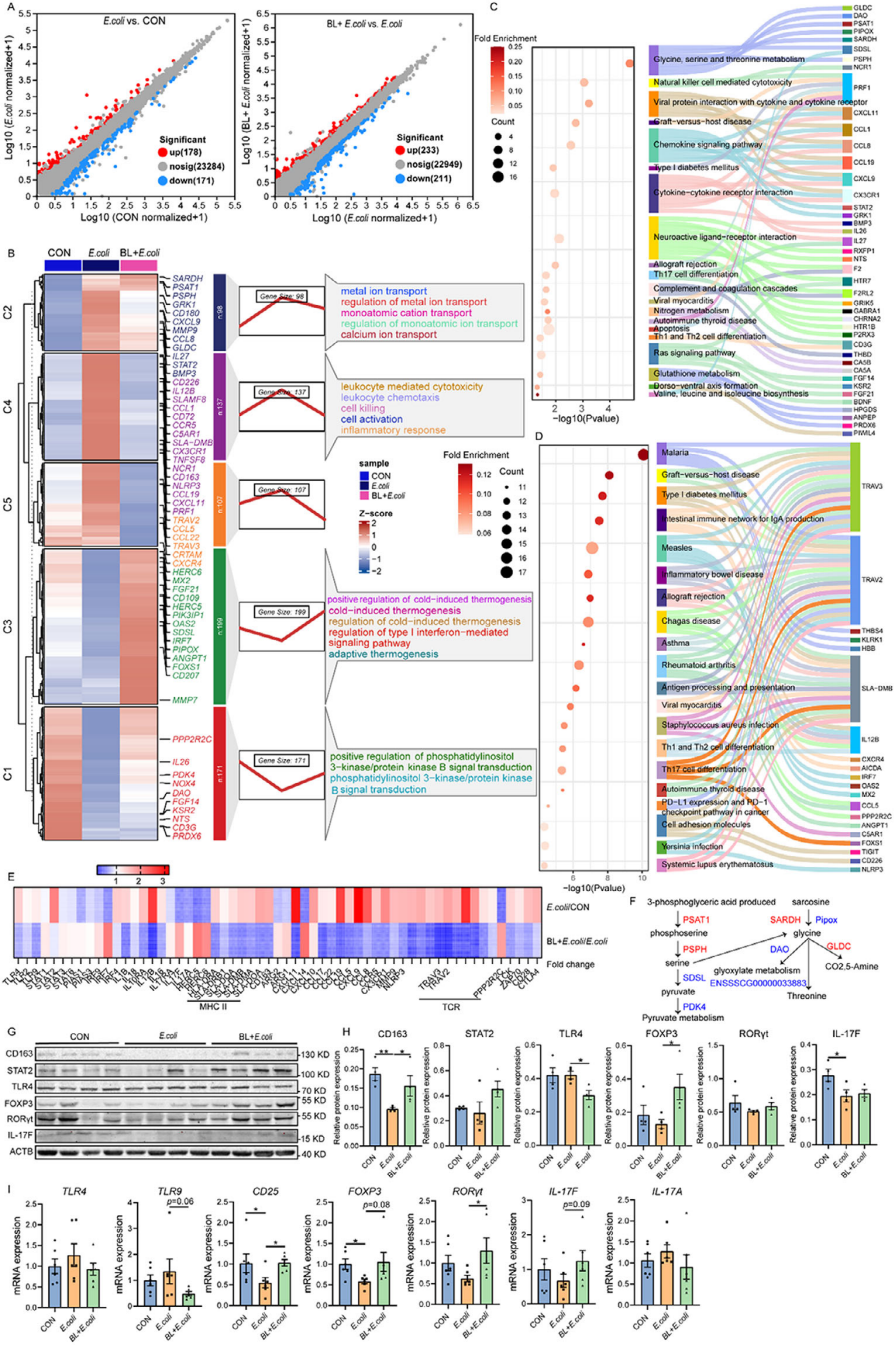
Baicalin regulates *E. coli*‐induced colon gene expression (*n* = 5). A) Scatter plot of expression difference (Fold change >1.8, *p* < 0.05). Each dot represents a gene, where red dots highlight significantly upregulated genes, blue dots indicate significantly downregulated genes, and gray dots represent genes with no significant differential expression. B) Clustering heat map, trend analysis and enriched GO terms for all differential genes in *E. coli* versus CON and BL + *E. coli* versus *E. coli* group. C) KEGG enrichment analysis of differential genes in *E. coli* versus CON; D) and BL + *E. coli* versus *E. coli* group. E) Fold change heatmap of key genes in Th17 cell differentiation pathways. F) Glycine‐threonine metabolism diagram. G) Western blotting was conducted to measure the expression of CD163, STAT2, TLR4, FOXP3, RORγt, IL‐17F, and ACTB in the colon, H) and the ratio of cleaved to full‐length forms for these proteins was calculated (*n* = 4). ACTB was used as a loading control. I) mRNA expression of *TLR4*, *TLR9*, *CD25*, *FOXP3*, *RORγt*, *IL‐17F*, and *IL‐17A* (*n* = 6). Data are presented as mean ± SEM and statistical analysis was performed using one‐way ANOVA followed by the LSD test. ^*^ means *p* < 0.05, ^**^ means *p* < 0.01.

RNA sequencing was performed to determine the molecular pathways through which baicalin alleviates intestinal injury and inflammation. The scatter plot displays the distribution of differentially expressed genes (DEGs) among groups (**Figure** **2**A). The expression patterns of these DEGs in the BL + *E. coli* group were aligned with those in the CON group as compared to the *E. coli* group (Figure 2B). Specifically, genes involved in metal ion transport (*SARDH*, *PSAT1*, *CD180*, *GRK1*, *IL27*, and *STAT2*), inflammatory response, cell killing pathways (*IL12B*, *CD226*, *CCL1*, *CCR5*, *CD163*, *NLRP3*, *CCL19*, *CXCL11*, and *PRF1*), and chemokines (*CCL5*, *CCL22*, and *CXCR4*) were increased in the *E. coli* group, however, decreased in the BL + *E. coli* group (*p* < 0.05, Figure 2B). However, genes involved in the regulation of type 1 interferon‐mediated signaling pathway (*MX2*, *FGF21*, *CD109*, *PIK3IPI*, *OAS2*, *IRF7*, *ANGPT1*, *PIPOX*, and *CD207*), and PI3K/AKT signaling (*PPP2R2C*, *IL26*, *PDK4*, *NOX4*, *DAO*, *FGF14*, *NTS*, and *PRDX6*) were downregulated in the *E. coli* group, however, upregulated in the BL + *E. coli* group (*p* < 0.05, Figure 2B).

Kyoto encyclopedia of genes and genomes (KEGG) pathway analysis revealed that DEGs in the *E. coli* versus CON group were primarily enriched in pathways associated with cytokine‐cytokine receptor interaction, chemokine signaling pathway, and Th17 cell differentiation (Figure 2C). In the BL + *E. coli* versus *E. coli* group, DEGs were enriched in immune modulation pathways, including those associated with malaria, inflammatory bowel disease, type 1 diabetes mellitus, measles, and Th1/Th2/Th17 cell differentiation (Figure 2D). Furthermore, T cell activation markers (*CD226* and *CRTAM*), lymphocyte activation molecules (*SLAMF7* and *SLAMF8*), and genes promoting Th17 cell differentiation (*IL27* and *IL26*) were upregulated in the *E. coli* group, however, downregulated in the BL+*E. coli* group (Figure 2D), suggesting that baicalin regulates the abnormal T cell differentiation induced by *E. coli*.

The phenotype of macrophages can influence their expression of costimulatory molecules (CD80 and CD86) or inhibitory molecules, including CTLA4, which regulates T cells' activation.^[^ ^24^ ^]^ Besides, genes expressed on the surface of macrophages, including *CD163*, *TLR9*, and *MHC II*, inflammatory factors including *IL‐1β*, *IL12B*, and *IL10RA*, chemokines (*CXCL11*, *CXCL14*, *CXCL10*, *CCL17*, *CCL22*, *CCL19*, *CCL5*, *CCL8*, and *CXCL9*), and the metalloprotease *MMP9*, were significantly upregulated in the *E. coli* group, suggesting that *E. coli* infection can induce macrophage polarization (Figure 2E). Genes involved in macrophage polarization, including *IRF7*, *STAT2*, *HERC5*, and *HERC6*, exhibited significant expression changes (*p* < 0.05, Figure 2E). Gene changes associated with glycine, serine, and threonine metabolism support this notion (Figure 2F). Under inflammatory conditions, M1 macrophages rapidly generate energy by enhancing glycolysis.^[^ ^25^, ^26^ ^]^ The upregulation of genes associated with glycine and serine synthesis pathways, including *PSAT1*, *PSPH*, *SARDH*, and *GLDC*, likely reflects the increased metabolic demands of M1 macrophage polarization, which requires increased anabolic activity to support immune cell proliferation during inflammation (*p* < 0.05, Figure 2F). Conversely, the downregulation of serine‐degrading enzyme *SDSL* and glycine‐degrading enzymes *DAO* and *PIPOX* helps maintain elevated levels of serine and glycine, facilitating anabolic metabolism (*p* < 0.05, Figure 2F).

Western blotting analysis revealed that baicalin supplementation increased CD163 and FOXP3 expression while inhibiting TLR4 expression (*p* < 0.05, Figure 2G,H). Furthermore, baicalin increased the mRNA expression of *CD25* (*p* < 0.05) and exhibited a trend toward inhibiting *TLR9* expression, while increasing the expression of *FOXP3*, *RORγt*, and *IL‐17F* (*p* < 0.1, Figure 2I). These results indicate that baicalin can regulate macrophage polarization and T cell differentiation.

### 2.3 Baicalin Inhibits LPS‐Induced M1 Macrophage Polarization In Vitro

**Figure 3:**
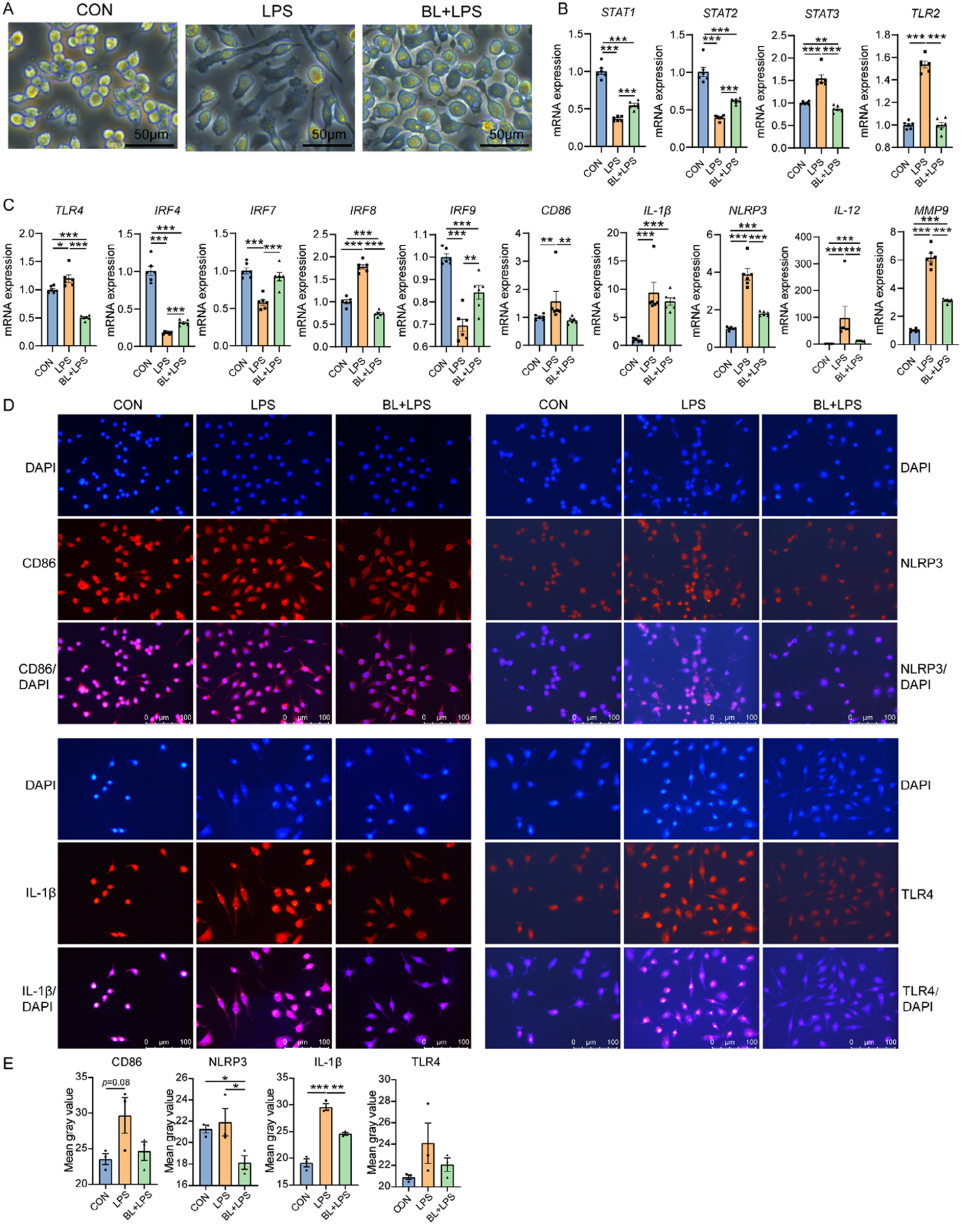
Baicalin inhibits LPS‐induced M1 macrophage polarization in vitro. A) Macrophages in CON, LPS, and BL + LPS group. B) mRNA expression of *STAT1*, *STAT2*, *STAT3*, and *TLR2* in CON, LPS, and BL + LPS group (*n* = 4). C) mRNA expression of *TLR4*, *IRF4*, *IRF7*, *IRF8*, *IRF9*, *CD86*, *IL‐1β*, *NLRP3*, *IL‐12*, and *MMP9* in CON, LPS, and BL + LPS group (*n* = 4). D) Immunofluorescence analyses of CD86, IL‐1β, NLRP3, and TLR4 protein expression in RAW264.7 cells. Nuclei are counterstained with DAPI (blue). (E) Mean gray value of CD86, IL‐1β, NLRP3, and TLR4 in immunofluorescence analyses (*n* = 3).

To further explore the effects of baicalin on macrophage polarization, RAW264.7 cells were treated with LPS (100 ng mL^−1^) for 12 h to induce M1 polarization. The CCK‐8 analysis indicated that baicalin at concentrations of 100, 200, and 300 µm concentrations restored macrophage viability following LPS treatment (Figure S1A, Supporting Information). Morphological analysis revealed that baicalin at 200 and 300 µm reversed the LPS‐induced morphological changes associated with M1 polarization (Figure S1B, Supporting Information). Therefore, 200 µm was selected for use in the following experiments. Baicalin significantly reversed the LPS‐induced morphological changes in macrophages (**Figure** **3**A).

Interferon regulatory factors (IRFs) are crucial in controlling macrophage maturation and phenotypic polarization.^[^ ^27^ ^]^ Specifically, IRF8 and IRF9 play key roles in promoting pro‐inflammatory M1 macrophage polarization, while IRF4 controls M2 polarization. IRF7 is involved in the transition between M1 and M2 phenotypes.^[^ ^28^, ^29^, ^30^ ^]^ Additionally, STAT factors that mediate cytokine and interferon signaling are closely associated with macrophage polarization.^[^ ^31^, ^32^ ^]^ However, compared to the LPS group, baicalin treatment upregulated the mRNA expression levels of *STAT1*, *STAT2*, *IRF4*, and *IRF7*, while downregulating the expression levels of *STAT3*, *TLR2*, *TLR4*, and *IRF8* (*p* < 0.05, Figure 3B,C). Moreover, baicalin decreased *CD86*, *IL‐1β*, *NLRP3*, *IL‐12*, and *MMP9* expression (*p* < 0.05, Figure 3C). Immunofluorescence analyses further confirmed that LPS stimulation upregulated the expression of M1 macrophage surface marker CD86 (*P* = 0.08) and pro‐inflammatory factors IL‐1β in RAW264.7 cells (*p* < 0.05, Figure 3D,E). However, baicalin treatment reduced the LPS‐induced upregulation of IL‐1β and NLRP3 (*p* < 0.05, Figure 3D,E). Moreover, the expression trend of TLR4 in each group was consistent with the mRNA level (Figure 3D,E). These findings suggest that baicalin inhibits LPS‐induced M1 macrophage polarization and reduces inflammation by regulating TLR4/IRF/STAT signaling pathway.

### 2.4 Baicalin Regulates the Colonic Microbial Composition

**Figure 4:**
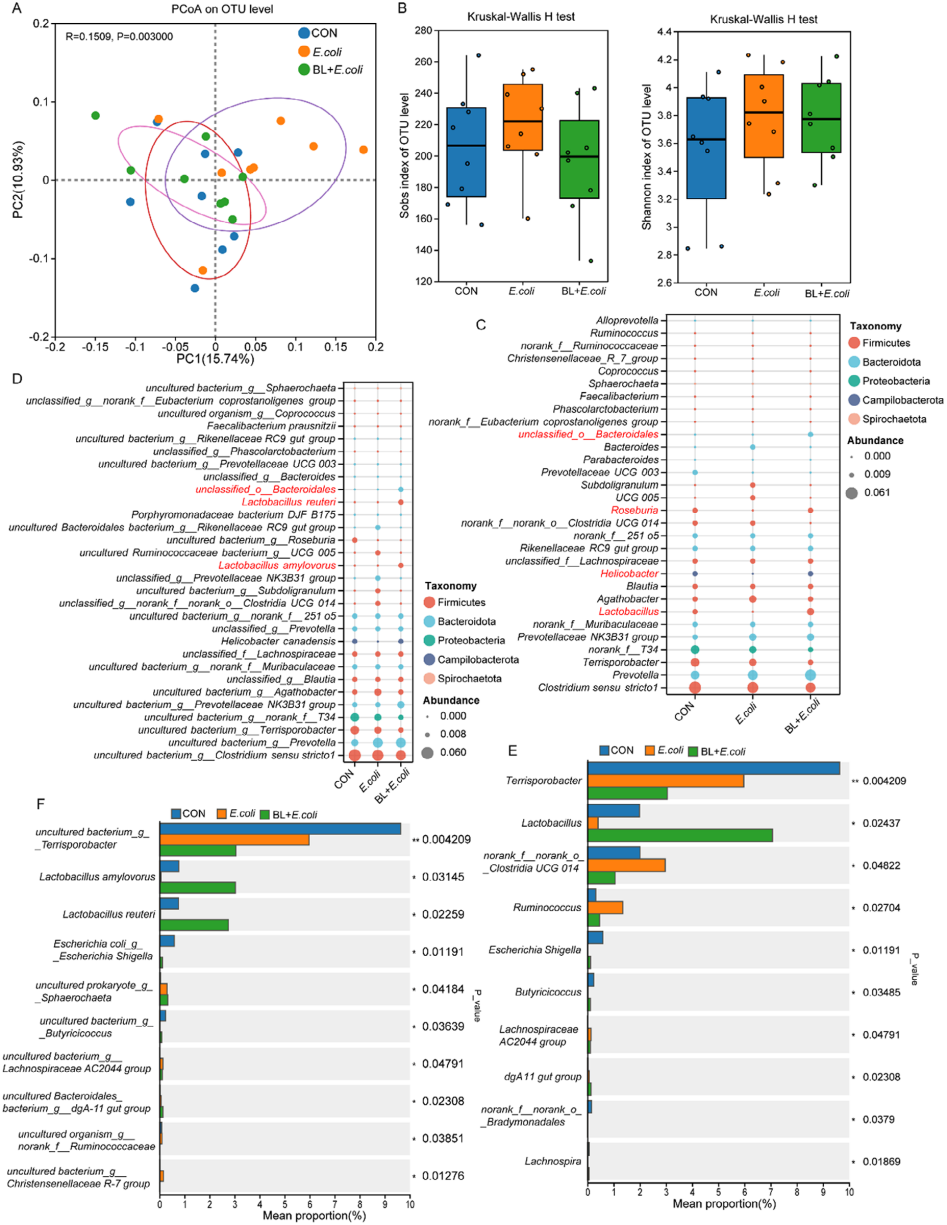
Baicalin regulates the colonic microbial composition (*n* = 8). A) PCoA analysis of microbiota in colon based on Weighted Unifrac distance metrics. B) Alpha‐diversity (Sobs and Shannon index) of microbiota. ^*^ means *p* < 0.05. C) Relative abundance of top 30 microbiota at genus level, D) and species level. E) Analysis of different microbiota among groups at genus level, F) and species level. The top 10 differential bacteria in abundance were shown. Intergroup differences were assessed using the Wilcoxon rank–sum test. ^*^ means *p* < 0.05, ^**^ means *p* < 0.01.

We investigated the characteristics of intestinal microbiome in piglets following treatment with baicalin and *E. coli*. Principal coordinate analysis (PCoA) demonstrated significant distinctions in bacterial profiles between the *E. coli* group and both CON and BL + *E. coli* groups (**Figure** **4**A). However, no significant differences were observed among groups regarding the Sobs and Shannon indices (Figure 4B), but the microbial composition differed (Figure 4C,D). At the genus level, the dominant bacterial genera in CON and *E. coli* group were *Clostridium sensu stricto1*, *Prevotella*, *Terrisporobacter*, and *norank_f__T34*, whereas in the BL + *E. coli* group, the major genera included *Lactobacillus, Prevotella*, *Prevotellaceae NK3B31 group*, and *Clostridium sensu stricto1* (Figure 4C). Comparative analysis exhibited a significant reduction in *Lactobacillus* abundance in the *E. coli* group compared to the CON group, with a significant increase observed following baicalin supplementation (*p* < 0.05, Figure 4E). Furthermore, *Ruminococcus* levels in the *E. coli* group significantly increased compared to the CON and BL + *E. coli* groups. At the species level, the abundance of *Lactobacillus amylovorus* and *Lactobacillus reuteri* were reduced in the *E. coli* group but increased in the BL + *E. coli* group (*p* < 0.05, Figure 4C,F). These fundings suggest that baicalin supplementation helps restore the intestinal microbial balance disrupted by *E. coli* infection and improving *Lactobacillus amylovorus* and *Lactobacillus reuteri* abundance.

### 2.5 Baicalin‐Mediated Enrichment of *Lactobacillus amylovorus* SKLAN202301ZF Alleviates *E. coli*‐Induced Intestinal Morphological Damage and Inflammation

**Figure 5:**
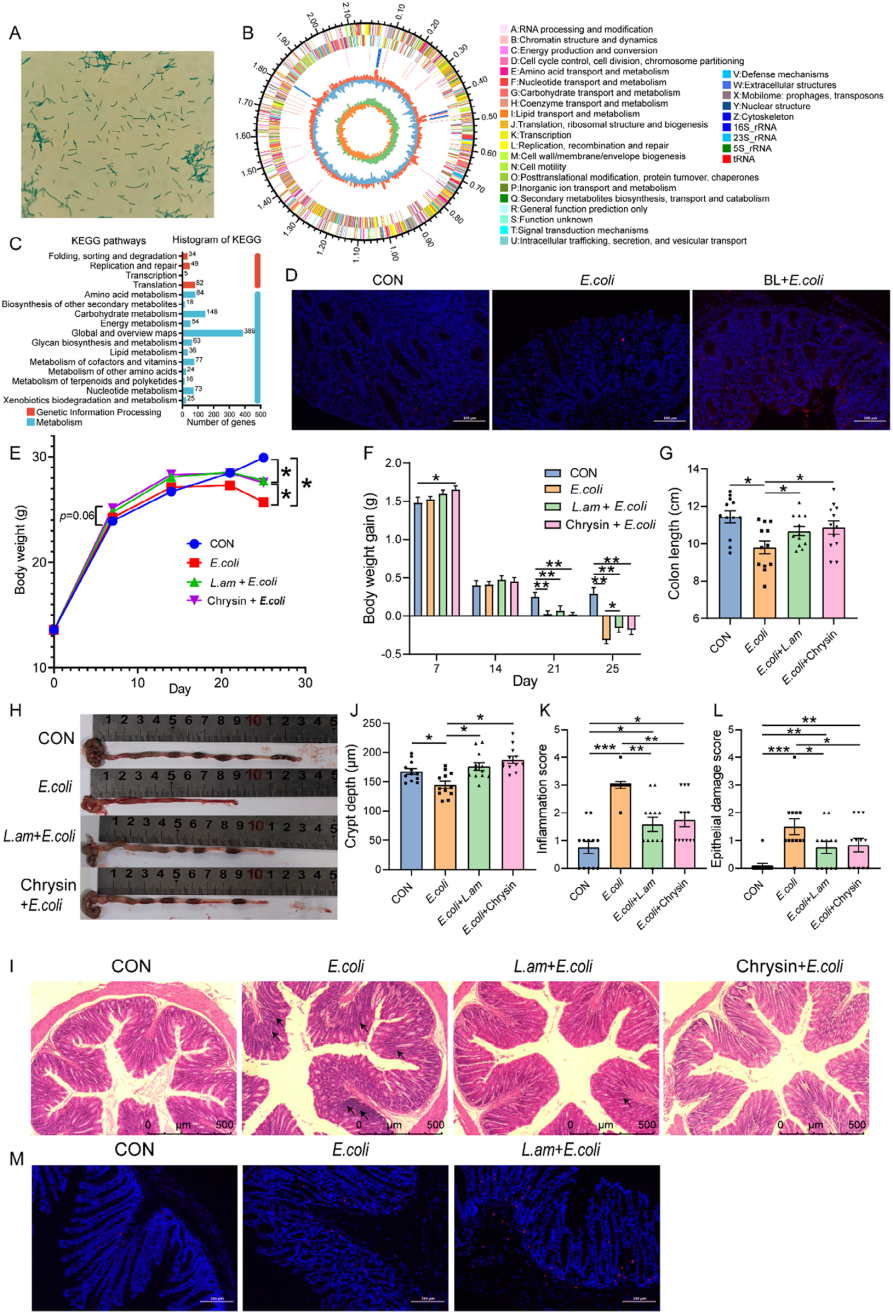
*Lactobacillus amylovorus* SKLAN202301ZF alleviates intestinal morphological damage and inflammation. A) The morphology of *Lactobacillus amylovorus* SKLAN202301ZF after malachite green staining under an oil immersion microscope. B) Circular genomic map of *Lactobacillus amylovorus* SKLAN202301ZF. The outermost circle of the circular diagram represents the genome size. The second and third circles represent the CDS on the positive and negative strands, with different colors indicating the COG functional categories of the CDS. The fourth circle represents rRNA and tRNA. The fifth circle shows the GC content, with the red sections pointing outward indicating regions with a higher GC content than the average GC content of the whole genome, and the blue sections pointing inward indicating regions with a lower GC content than the average GC content. The innermost circle represents the GC‐skew value, calculated as (G‐C)/(G+C). C) KEGG pathway annotation classification statistics of *Lactobacillus amylovorus* SKLAN202301ZF. D) Spatial location of *Lactobacillus amylovorus* SKLAN202301ZF in the piglet colon was examined using fluorescent in situ hybridization (FISH). Bacteria are shown in red, and DNA in blue. Scale bars = 200 µm. E) Body weight (*n* = 12). F) Body weight gain (*n* = 12). G) Colon length (*n* = 12). H) Colonic morphology. I) Staining profiles by H&E of colon (scale bars: 500 µm). J) Colonic crypt depth (*n* = 12). K) Colonic inflammation score (*n* = 12). L) Colonic epithelial damage score (*n* = 12). M) Spatial location of Lactobacillus amylovorus SKLAN202301ZF in the piglet colon was examined using fluorescent in situ hybridization (FISH). Bacteria are shown in red, and DNA in blue. Scale bars = 200 µm.

To investigate the role of *Lactobacillus amylovorus* in alleviating *E. coli*‐induced inflammation, we isolated a strain of *Lactobacillus amylovorus* SKLAN202301ZF from piglets in the BL + *E. coli* group (**Figure** **5**A). Further analysis through whole‐genome sequencing revealed the presence of the *β*‐glucuronidase gene in the genome of *Lactobacillus amylovorus* SKLAN202301ZF that can hydrolyze baicalin into the small molecule baicalein (Figure 5B; Table S8, Supporting Information). Gene annotation analysis revealed that multiple genes in *Lactobacillus amylovorus* SKLAN202301ZF are involved in carbohydrate and amino acid metabolism (Figure 5C). To determine the spatial distribution of *Lactobacillus amylovorus* SKLAN202301ZF in the colon, we performed fluorescent in situ hybridization (FISH). The piglets in the BL + *E. coli* group exhibited higher abundance of *Lactobacillus amylovorus* SKLAN202301ZF (as indicated by red fluorescence) than those in CON and *E. coli* groups (Figure 5D).

We further performed a supplementation experiment in mice to demonstrate the beneficial effects of *Lactobacillus amylovorus* SKLAN202301ZF on intestinal inflammation. The body weight and weight gain in the *L. am + E. coli* group were significantly higher than those in the *E. coli* group (*p *< 0.05, Figure 5E,F). Additionally, mice in the *L. am* + *E. coli* group exhibited significant increases in colon length and crypt depth compared to the *E. coli* group (*p *< 0.05, Figure 5G,H,J), while inflammation and epithelial damage scores were significantly lower (*p *< 0.05, Figure 5I,K,L). Spatial location analysis of *Lactobacillus amylovorus* SKLAN202301ZF in the colon exhibited significant abundance in the *L. am* + *E. coli* group (Figure 5M). These results suggest that *Lactobacillus amylovorus* can alleviate *E. coli*‐induced intestinal inflammation.

### 2.6 *Lactobacillus amylovorus* SKLAN202301ZF Inhibites TLR/IRF/STAT Pathway to Regulate *E. coli*‐Induced Macrophage Polarization and Inflammation

**Figure 6:**
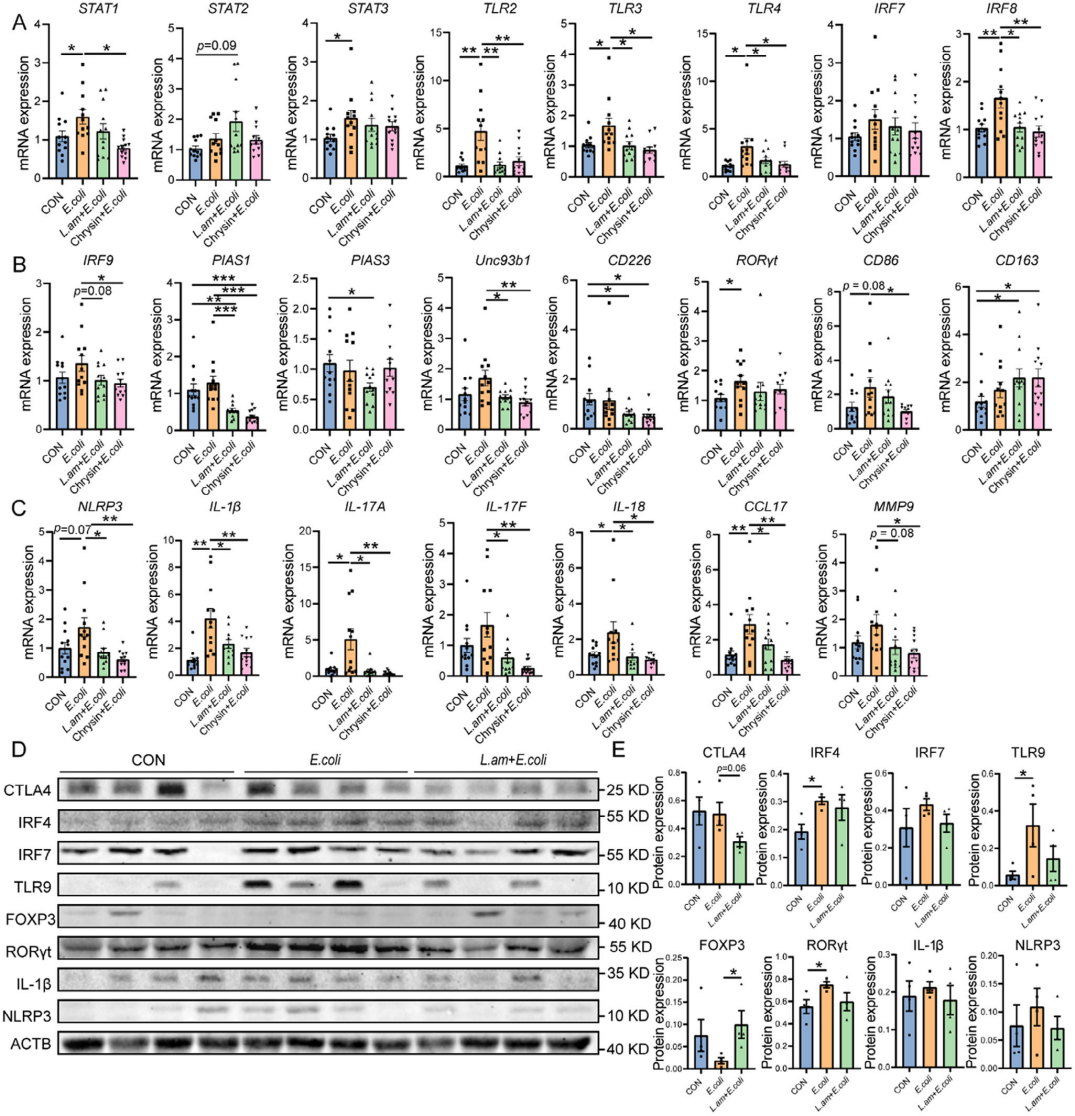
*Lactobacillus amylovorus* SKLAN202301ZF regulates macrophage polarization and T cell differentiation. A) mRNA expression of *STAT1*, *STAT2*, *STAT3*, *TLR2*, *TLR3*, *TLR4*, *IRF7*, and *IRF8* (*n* = 12). B) mRNA expression of *IRF9*, *PIAS1*, *PIAS3*, *CD226*, *Unc93b1*, *RORγt*, *CD86*, and *CD163* (*n* = 12). C) mRNA expression of *NLRP3*, *IL‐1β*, *IL‐17A*, *IL‐17F*, *IL‐18*, *CCL17*, *MMP9* (*n* = 12). D) Western blotting was conducted to measure the expression of CTLA4, IRF4, IRF7, TLR9, FOXP3, RORγt, IL‐1β, NLRP3, and ACTB in the colon, E) and the ratio of cleaved to full‐length forms for these proteins was calculated (*n* = 4). ACTB was used as a loading control.

The mechanism by which *Lactobacillus amylovorus* SKLAN202301ZF alleviates intestinal inflammation was further investigated. Compared to the *E. coli* group, *Lactobacillus amylovorus* SKLAN202301ZF significantly inhibited the mRNA expression levels of *TLR2*, *TLR3*, *TLR4*, *IRF8*, *IRF9*, and the TLR signaling regulator *Unc93b1* (*p* < 0.05, **Figure** **6**A,B). Besides, it reduced the expression of *PIAS1* and *PIAS3*, which are inhibitors of *STAT1* and *STAT3* (*p* < 0.05, Figure 6B). Moreover, *Lactobacillus amylovorus* SKLAN202301ZF decreased the expression of the T cell activation marker *CD226*, the Th17 cell transcription factor *RORγt*, and *CD86*, while increasing *CD163* expression (*p* < 0.05, Figure 6B). *Lactobacillus amylovorus* decreased *NLRP3*, *IL‐1β*, *IL‐17A*, *IL‐17F*, *IL‐18*, *CCL17*, and *MMP9* levels (*p* < 0.05, Figure 6C). Western blotting analysis further demonstrated that *Lactobacillus amylovorus* SKLAN202301ZF decreased the expression of T cell antigen‐presenting receptors *CTLA4* and *RORγt* while increasing *FOXP3* expression (*p* < 0.05, Figure 6D,E). These fundings suggest that *Lactobacillus amylovorus* SKLAN202301ZF regulates macrophage polarization and T cell differentiation and contributes to alleviating intestinal inflammation through TLR/IRF/STAT pathway.

### 2.7 *Lactobacillus amylovorus* SKLAN202301ZF Inhibits LPS‐Induced M1 Macrophage Polarization In Vitro

**Figure 7:**
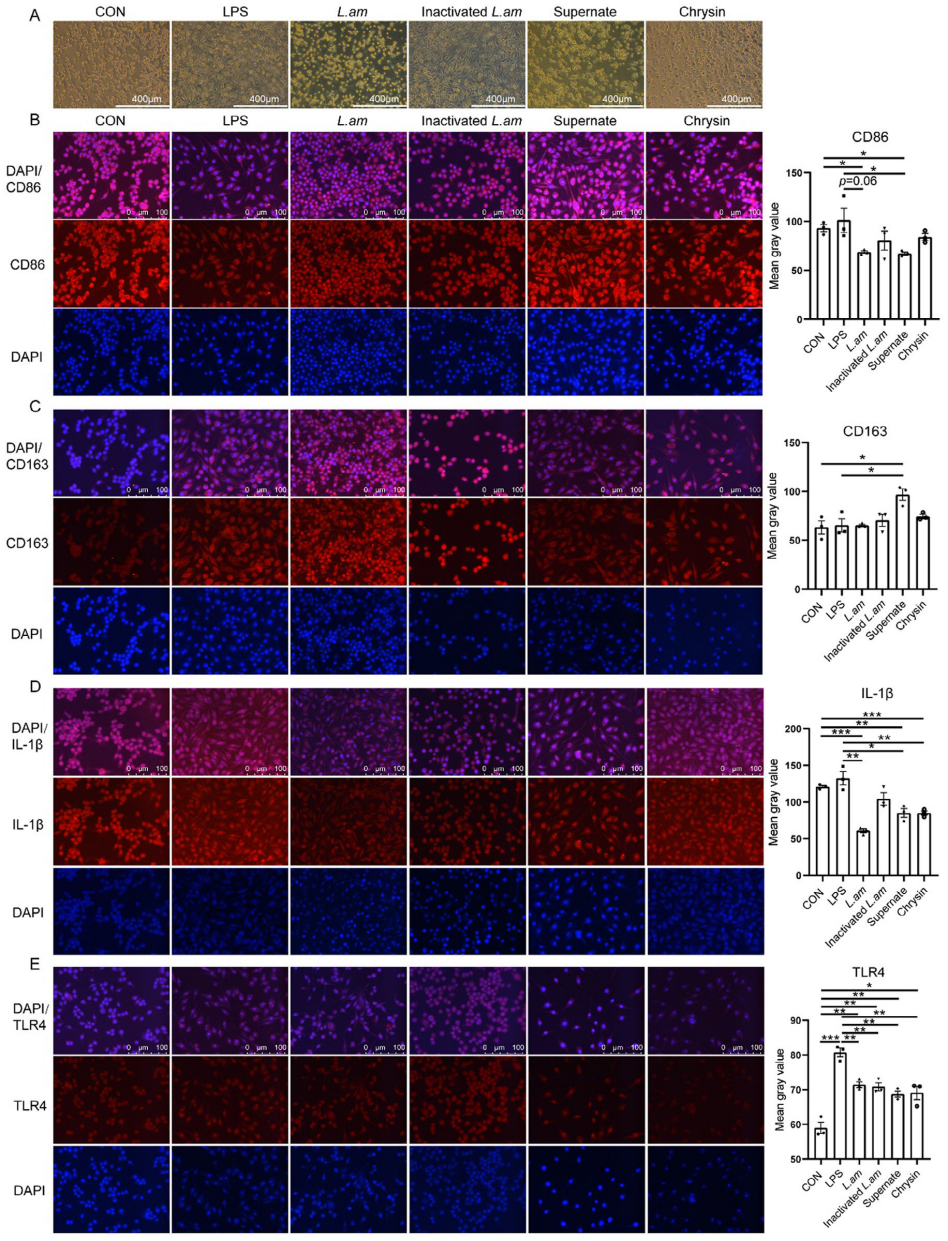
*Lactobacillus amylovorus* SKLAN202301ZF and chrysin regulates macrophage polarization in vitro (*n* = 3). A) Cellular morphology of RAW264.7 cells in each group. B) Immunofluorescence analyses and mean gray value of CD86, C) CD163, D) IL‐1β, and E) TLR4 protein expression in Raw264.7 cells. Nuclei are counterstained with DAPI (blue).

To further explore the effects of *Lactobacillus amylovorus* SKLAN202301ZF on macrophage polarization, RAW264.7 cells were treated with LPS (100 ng mL^−1^) for 12 h to induce M1 polarization. After LPS treatment, macrophages exhibited a spindle shape with protrusions and a larger size (**Figure** **7**A). However, after treatment with *Lactobacillus amylovorus* SKLAN202301ZF or its culture supernatant, the macrophages reverted to a round shape and reduced size (Figure 7A). Heat‐inactivated *Lactobacillus amylovorus* SKLAN202301ZF did not affect macrophage morphology (Figure 7A). Immunofluorescence analysis demonstrated that, compared to the LPS group, *Lactobacillus amylovorus* SKLAN202301ZF and its culture supernatant inhibited CD86 expression (*p* < 0.05, Figure 7B), while the latter also promoted CD163 expression (*p* < 0.05, Figure 7C). However, heat‐inactivated *Lactobacillus amylovorus* SKLAN202301ZF did not affect the expression of CD86 or CD163 (*p* > 0.05, Figure 7B,C). *Lactobacillus amylovorus* SKLAN202301ZF and its culture supernatant reduced IL‐1β and TLR4 expression compared to the LPS group (*p* < 0.05, Figure 7D,E). Heat‐inactivated *Lactobacillus amylovorus* SKLAN202301ZF had no significant effect on IL‐1β expression (*p* > 0.05, Figure 7D) but inhibited TLR4 expression (*p* < 0.05, Figure 7E). These fundings suggest that *Lactobacillus amylovorus* SKLAN202301ZF or its metabolites can regulate macrophage polarization and inhibit LPS‐induced inflammation.

### 2.8 *Lactobacillus amylovorus* SKLAN202301ZF May Exert Anti‐Inflammatory Effects Through its Metabolites

**Figure 8:**
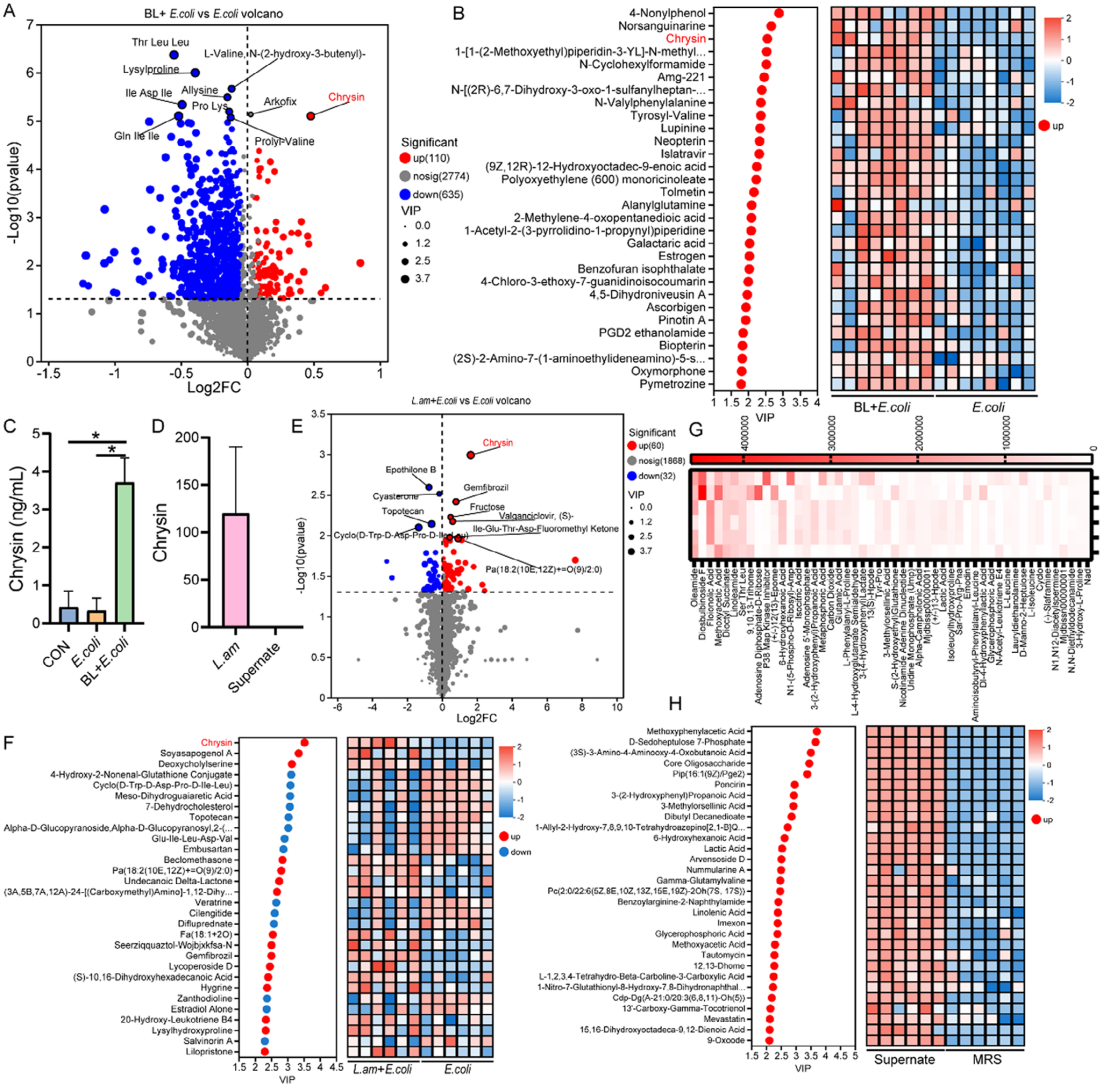
Baicalin regulates colonic metabolism disrupted by *E. coli* infection (*n* = 6). A) Volcano of differential metabolites in BL + *E. coli* versus *E. coli* of the piglets. Each dot corresponds to a single metabolite. Red dots indicate metabolites that are significantly upregulated, blue dots represent those that are significantly downregulated, and gray dots denote metabolites with no significant differential expression. B) Expression profile and VIP analysis of metabolites for BL + *E. coli* and *E. coli* group of the piglets. C) LC‐MS analysis of chrysin in piglets. D) Chrysin content in *Lactobacillus amylovorus* SKLAN202301ZF and its culture. E) Volcano of differential metabolites in *L. am* + *E. coli* versus *E. coli* of the mice. F) Expression profile and VIP analysis of metabolites for *L. am* + *E. coli* and *E. coli* group of the mice. G) Top 50 metabolites in *Lactobacillus amylovorus* SKLAN202301ZF culture. H) Expression profile and VIP analysis of metabolites for *Lactobacillus amylovorus* SKLAN202301ZF culture and MRS broth medium.

Untargeted metabolomics was performed to investigate whether *Lactobacillus amylovorus* SKLAN202301ZF exerts anti‐inflammatory effects through its metabolites. In the colonic chyme of piglets from the BL + *E. coli* group, 110 metabolites, including chrysin, indoles, organic acids, and amines, exhibited increased levels compared to the *E. coli* group (**Figure** **8**A; Table S5, Supporting Information). However, 635 metabolites, primarily upregulated in the *E. coli* versus CON group (Figure S2A, Supporting Information), were significantly downregulated in BL + *E. coli* versus *E. coli* group. VIP analysis identified the top 10 upregulated metabolites in the BL + *E. coli* group, including 4‐nonylphenol, norsanguinarine, chrysin, N‐cyclohexylformamide, Amg‐221, N‐valylphenylalanine, and Tyrosyl‐Valine (Figure 8B). Additionally, chrysin exhibited the highest VIP values among the differential metabolites in the BL + *E. coli* versus CON comparison (Figure S2B, Supporting Information). Chrysin levels in the colonic chyme of piglets from all three groups were quantified using liquid chromatography‐mass spectrometry with a chrysin standard. The results supported the untargeted metabolomics findings, suggesting that the chrysin levels in the BL + *E. coli* group were significantly elevated compared to those in the CON and *E. coli* groups (*p* < 0.05, Figure 8C). Additionally, chrysin was detected in vitro cultured *Lactobacillus amylovorus* SKLAN202301ZF strain, although no chrysin was observed in the culture supernatant (Figure 8D; Figure S2C, Supporting Information). In the *Lactobacillus amylovorus* SKLAN202301ZF supplementation experiment, chrysin was identified as the most significantly different metabolite in the colonic chyme of mice in the *L. am* + *E. coli* group compared to the *E. coli* group (Figure 8E; Table S6, Supporting Information), with the highest VIP value (Figure 8F). Furthermore, metabolites including amino acids, organic acids and bile acids were significantly upregulated after *Lactobacillus amylovorus* SKLAN202301ZF supplementation (*p* < 0.05, Table S7, Supporting Information).

We then validated the effect of chrysin on alleviating intestinal inflammation. Chrysin supplementation for one week increased the body weight (*P* = 0.06, Figure 5E), with significant gains in both body weight and colon length observed compared to the *E. coli* group (*p* < 0.05, Figure 5F–H). Histological analysis through hematoxylin and eosin staining revealed that chrysin improved colonic villus morphology, significantly increased the depth of colonic crypts, and reduced inflammation and epithelial damage scores (*p* < 0.05, Figure 5I–L). Moreover, compared to the *E. coli* group, chrysin supplementation significantly reduced the expression levels of *STAT1*, *TLR2*, *TLR3*, *TLR4*, *IRF8*, *IRF9*, *PIAS1*, *Unc93b1*, and *CD86* (*p* < 0.05, Figure 6A,B). Chrysin decreased *NLRP3*, *IL‐1β*, *IL‐17A*, *IL‐17F*, *IL‐18*, *CCL17*, and *MMP9* levels compared to the *E. coli* group (*p* < 0.05, Figure 6C). In the RAW264.7 cell experiment, chrysin mitigated LPS‐induced morphological changes in macrophages (Figure 7A) and inhibited TLR4 and IL‐1β expression (*p* < 0.05, Figure 7D,E). These results suggest that one of the possible pathways through which *Lactobacillus amylovorus* SKLAN202301ZF alleviates intestinal inflammation is the synthesis of chrysin, which inhibits TLR expression, thereby suppressing macrophage activation and antigen presentation.

In addition, in the untargeted metabolomics analysis of both *Lactobacillus amylovorus* SKLAN202301ZF and its culture supernatant, we identified potential metabolites, including methoxyphenylacetic acid, 3‐(2‐hydroxyphenyl) propanoic acid, floionolic acid, 3‐methylorsellinic acid, lycoperoside D, lactic acid, 6‐hydroxyhexanoic acid, linolenic acid, and glycerophosphoric acid (Figure 8G,H). Metabolites, including amines (spermidine, methoxamine, N1‐acetylspermidine), bile acids (taurocholic acid, 7‐ketolithocholic acid, deoxycholic acid), organic acids (phaseolic acid, gamma‐linolenic acid, carboxylic acid, floionolic acid) and lactic acid were upregulated in the culture supernatant (*p* < 0.05, Table S7, Supporting Information). However, further investigation is required to determine whether these metabolites contribute to the anti‐inflammatory function of *Lactobacillus amylovorus* SKLAN202301ZF.

## 3 Discussion

This study explored the mechanisms through which baicalin alleviates bacterial intestinal inflammation. The results revealed that baicalin directly regulates macrophage polarization and T cell differentiation to reduce inflammatory responses. Furthermore, it increases *Lactobacillus amylovorus* abundance and the synthesis of its metabolite, chrysin, to alleviate intestinal inflammation. Previous studies have shown that the anti‐inflammatory effects of baicalin are primarily attributed to its ability to inhibit cyclooxygenase‐1, cyclooxygenase‐2, and 5‐lipoxygenase,^[^ ^33^ ^]^ regulate the TLR4/NF‐κB/MAPK signaling pathway,^[^ ^12^, ^34^ ^]^ and suppress inflammasome activation and the secretion of inflammatory cytokines.^[^ ^35^ ^]^ Besides, baicalin has been reported to regulate macrophage polarization and T cell activation through the JAKs/AMPK signaling pathway.^[^ ^36^ ^]^ Previous studies have reported a correlation between macrophage size and phenotype, where larger macrophages exhibit a pro‐inflammatory profile, while smaller macrophages are more likely to display an anti‐inflammatory phenotype.^[^ ^37^, ^38^, ^39^ ^]^ In this study, the macrophage volume significantly decreased following baicalin supplementation, suggesting its role in inhibiting M1 macrophage polarization. Baicalin also reduced M1 macrophage polarization and inflammatory cytokines (*IL‐1β*, *IL‐12*, and *IL‐18*) secretion by suppressing the expression of *TLR4*, *TLR2*, and *TLR3* on macrophage surfaces. These results consistent with the study which demonstrated that baicalin inhibits TLR4 activation and subsequent inflammation by downregulating CD14, a co‐receptor of LPS.^[^ ^34^ ^]^ Moreover, baicalin regulates the expression of key genes in macrophage polarization, including *IRF7*, *IRF4*, *IRF8*, *IRF9*, *STAT1*, *STAT3*, and *STAT2*, which aligns with earlier studies indicating that baicalin promotes M1/M2 balance by decreasing *IRF5* expression and increasing *IRF4* protein levels.^[^ ^36^, ^40^ ^]^ The macrophage phenotype can influence antigen presentation and T cell activation.^[^ ^41^, ^42^ ^]^ In this study, we observed an upregulation of Treg‐associated genes and a downregulation of Th17‐related genes after baicalin treatment. This aligns with previous findings that baicalin inhibits Th17 cell differentiation in the spleen to alleviate arthritic symptoms.^[^ ^43^ ^]^ Multiple natural flavonoids exert anti‐inflammatory effects by regulating the Th17/Treg balance through key transcription factors, including *RORγt*, *STAT3*, *FOXP3*, and *STAT5*, as well as signaling pathways including AhR, mTOR, and PI3K/Akt.^[^ ^44^ ^]^ Alpinetin and baicalein function as AhR agonists to promote Treg differentiation,^[^ ^45^, ^46^ ^]^ while tangeretin and icariin alleviate colitis symptoms by inhibiting the Notch1/Jagged1 and *STAT3* pathways, respectively, thereby reducing Th17 cell differentiation. In contrast to many of these flavonoids, baicalin not only directly influences Th17/Treg cell differentiation but also exerts its immunomodulatory effects through gut microbiota regulation.

Existing studies have indicated that baicalin can inhibit the growth of pathogenic bacteria, including *Staphylococcus aureus*, *E. coli*, and *Candida albicans*, while also promoting the abundance of probiotics, including those that produce short‐chain fatty acids.^[^ ^17^, ^47^, ^48^ ^]^ Baicalin alleviates periodontitis and lung injury by increasing the abundance of beneficial bacteria, including *Butyricicoccus*, *Alloprevotella*, *Blautia*, and *Akkermansia*.^[^ ^49^ ^]^ In this study, baicalin increased the abundance of the potential probiotics, *Lactobacillus amylovorus*, *Lactobacillus reuteri*, and *unclassified_o_Bacteroidates*, and alleviates intestinal inflammation. This may be attributed to the antibacterial effect of baicalin, which inhibits the growth of pathogenic bacteria, thereby reducing competition for nutrients and ecological niches, and creating a gut environment that favors the growth of beneficial bacteria. Another possible reason is that baicalin restored the number of goblet cells in the colon, ensuring mucus secretion, which provides healthy attachment sites and sufficient nutrients for colonizing beneficial bacteria.^[^ ^50^ ^]^ *Lactobacillus amylovorus* was a potential probiotic frequently used in animal husbandry as a silage fermentation additive,^[^ ^51^, ^52^ ^]^ which can degrade cellulose and lignin in silage, increasing the content of soluble carbohydrates.^[^ ^53^ ^]^ Studies have suggested that *Lactobacillus amylovorus* can regulate intestinal microbial composition and enhance intestinal barrier function.^[^ ^23^, ^54^ ^]^ Wu et al.,^[^ ^55^ ^]^ found that *Lactobacillus amylovorus* and its metabolite, lactic acid, enhance the growth and differentiation of intestinal stem cells through the Wnt/*β*‐catenin pathway. Gut microbiota is widely recognized as a key regulator of intestinal innate and adaptive immune responses.^[^ ^56^, ^57^ ^]^ *Lactobacillus species*, including *Lactobacillus acidophilus*, *Lactobacillus paracasei*, *Lactobacillus plantarum*, and *Lactobacillus reuteri*, have been reported to reduce Th17 cell differentiation and promote Treg cell differentiation.^[^ ^58^, ^59^, ^60^ ^]^ Studies have demonstrated that *Lactobacillus amylovorus* suppresses ETEC‐induced increases in *IL‐1β* and *IL‐18* by inhibiting *TLR4* signaling and *MyD88* expression in in vitro epithelial cell models.^[^ ^61^ ^]^ Consistent with these findings, our study revealed that *Lactobacillus amylovorus* significantly suppresses the expression of *TLR2*, *TLR3*, and *TLR4*, along with downstream mediators including *PIAS1*, *IRF8*, *IRF9*, and the TLR signaling regulator *Unc93b1*, thereby alleviating *E. coli*‐induced intestinal inflammation. These findings suggest that *Lactobacillus amylovorus* may exerts anti‐inflammatory effects via TLR/IRF/STAT pathway, a mechanism that has not yet been reported in similar in vivo studies. Notably, *Lactobacillus amylovorus* may also exert its beneficial effects by metabolizing baicalin. In this study, *β*‐glucuronidase was found in the genome of this strain for the first time (Table S8, Supporting Information), a key enzyme driving baicalin metabolism, which catalyzing the deglycosylation of flavonoids, thereby enhancing their bioactivity.^[^ ^20^ ^]^

Chrysin is a flavonoid that has received significant attention as one of the most important bioactive constituents in various fruits, vegetables, and mushrooms.^[^ ^62^, ^63^ ^]^ Chrysin has been reported to possess liver protective, anti‐inflammatory, antioxidant, and immunomodulatory functions.^[^ ^64^, ^65^ ^]^ Additionally, it can reduce inflammatory factors and alleviate rheumatoid arthritis.^[^ ^66^ ^]^ Chrysin has been reported to alleviate inflammatory responses by inhibiting the TLR/TRIF pathway.^[^ ^67^ ^]^ This study further revealed that chrysin may alleviate inflammation by suppressing the expression of the TLR modulator *Unc93b1* and downregulating *STAT1*, *IRF8*, and *IRF9*, thereby regulate TLR/IRF/STAT signaling pathway. In this study, chrysin were detected in the colonic chyme of piglets received baicalin, and *Lactobacillus amylovorus* SKLAN202301ZF, as well as in mice that received *Lactobacillus amylovorus* SKLAN202301ZF, suggesting a potential association between *Lactobacillus amylovorus* SKLAN202301ZF and chrysin presence, though direct evidence of its synthesis is lacking. There are two possible pathways for the formation of chrysin. One pathway involves the conversion of baicalin into baicalein by microbial *β*‐glucuronidase, followed by a decarboxylation reaction mediated by microbiota. The phenolic acid decarboxylase gene detected in the genome of *Lactobacillus amylovorus* may participate in this process (Table S9, Supporting Information). The ability of *Lactobacillus* species and other genera to regulate the transformation of flavonoid compounds has been well documented.^[^ ^68^, ^69^ ^]^ Another potential source of chrysin may rely on the metabolic synthesis by a microbial community centered around *Lactobacillus amylovorus*. The shikimate pathway could play a role in this process, resulting in the sequential synthesis of cinnamic acid, chalcone, and naringenin, ultimately producing chrysin.^[^ ^70^, ^71^ ^]^ This process involves complex metabolic pathways and interactions among microbial communities. Although there is currently no direct evidence to fully elucidate this synthetic process, we have identified key enzyme genes in the shikimate pathway within the genome of *Lactobacillus amylovorus* (Table S9, Supporting Information). Although chrysin was undetected in the supernatant of *Lactobacillus amylovorus* SKLAN202301ZF cultured in vitro, this may be due to host‐derived enzymes or cross‐feeding interactions with commensal microbiota may also participate in this metabolic cascade. Further research is required to support this hypothesis. Metabolites such as lactic acid, indole, spermidine and bile acid, previously identified as potential anti‐inflammatory agents,^[^ ^72^, ^73^, ^74^ ^]^ were upregulated in the intestines of mice supplemented with *Lactobacillus amylovorus* and its in vitro supernatant. Lactic acid in the intestinal microenvironment has been reported to promote histone *H3K27* acetylation, facilitating *Nr4a1* expression and thereby suppressing the pro‐inflammatory response of macrophages.^[^ ^75^ ^]^ Microbiota‐derived indoles could alleviate inflammation through microbial cross‐feeding by increasing the abundance of tryptophan‐metabolizing bacteria and enhancing the expression of acyl‐CoA dehydrogenase and indolelactate dehydrogenase.^[^ ^76^ ^]^ Additionally, supplementation with spermidine promotes CD4^+^ T cell differentiation into Treg cells while reducing IL‐17 levels, ultimately alleviating colonic inflammation.^[^ ^73^ ^]^ Bile acids further contribute to immune modulation by binding to RORγt, thereby inhibiting Th17 cell differentiation and activating FOXP3 to promote Treg cell differentiation.^[^ ^77^ ^]^ However, whether *Lactobacillus amylovorus* SKLAN202301ZF exerts its anti‐inflammatory effects through these metabolites requires further investigation.

In conclusion, this study systematically explores the mechanisms by which baicalin alleviates bacterial intestinal inflammation from both its direct effects and the microbiota‐mediated indirect effects. It is the first to propose the critical role of the *Lactobacillus amylovorus* and chrysin in the anti‐inflammatory mechanism of baicalin and highlight that *Lactobacillus amylovorus* not only functions as a probiotic but may also serves as a key strain in baicalin metabolism, providing a novel explanation for its anti‐inflammatory effects. This study provides new insights for the future development of baicalin, *Lactobacillus amylovorus*, and their metabolites in the prevention and treatment of bacterial diarrhea, traveler's diarrhea, and inflammatory bowel disease. There are several limitations in this study. A key area for future research is a more detailed understanding of the mechanisms through which *Lactobacillus amylovorus* produces chrysin. While we have demonstrated the production of chrysin, the full metabolic pathways, biosynthetic intermediates, and the enzymatic processes involved in its synthesis remain unclear. Current methods do not yet allow us to identify specific precursor molecules or elucidate the complete enzymatic processes involved. Addressing these gaps will be crucial for optimizing baicalin's therapeutic application in clinical settings. Future studies using advanced metabolomics and genetic tracing methods must adequately characterize this mechanism.

## 4 Experimental Section

### Experiment Design

Weaned piglets (Duroc‐Landrace‐Yorkshire; initial weight: 6.19  ±  0.51 kg; 24 days old; half male and half female) were evenly distributed into three groups based on their weight: CON, *E. coli*, and BL + *E. coli*, each comprising eight piglets. The piglets in the CON and *E. coli* groups were given a basal diet, while those in the BL + *E. coli* group were provided with a basal diet supplemented with 100 mg kg^−1^ of baicalin (purity ≥ 85%, Peking Centre Technology Co., LTD., Beijing, China). All piglets had unlimited access to food and water and kept in a well‐maintained and comfortable environment. On days 7, 10, and 15 of the experiment, piglets in the *E. coli* and BL + *E. coli* group were intraperitoneally injected with 1, 2, and 3 mL of *E. coli* SKLAN202302 (concentration: 2 × 10^9^ CFU mL^−1^), respectively. Piglets in the CON group were intraperitoneally injected with an equal amount of normal saline. The *E. coli* SKLAN202302 strain was isolated from the colonic contents of diarrheal piglets in our laboratory and stored at the China General Microbiological Culture Collection Center under storage number CGMCC No.26420. After the experiment, the piglets were humanely euthanized, and the colonic mucosa and contents were harvested for further analysis. All experimental procedures and animal care protocols received approval from the Institutional Animal Care and Use Committee at the Institute of Animal Science, Chinese Academy of Agricultural Sciences (Approval number: IAS2022‐113).

### Hematoxylin and Eosin (H&E) and Alcian Blue and Periodic Acid Schiff (AB‐PAS) Staining

After collection, the colon samples were immersed in Carnoy's fixative (a mixture of 60% methanol, 30% chloroform, and 10% acetic acid) for a minimum of 24 h. Following fixation, the tissues were dehydrated, infiltrated with paraffin, and sectioned for subsequent analysis. H&E staining was used to examine intestinal morphology and determine crypt depth. Additionally, goblet cell counts were determined using AB‐PAS staining, according to the manufacturer's protocol (Solarbio, Beijing, China). All measurements were performed on stained slides using a DM300 light microscope (Leica, Germany). Histological specimens were randomized and scored blindly by a board‐certified veterinary pathologist. The epithelial damage scoring system was performed as Table S1 (Supporting Information) and the inflammation scoring system was defined as Table S2 (Supporting Information).

### Transcriptome Analysis

Colonic mucosa samples were collected for total RNA extraction using the phenol‐chloroform method. The construction and sequencing of the cDNA library were performed by Majorbio Bio‐Pharm Technology Co. Ltd. (Shanghai, China). RNA samples were considered high‐quality based on the following criteria: OD260/280 between 1.8 and 2.2, OD260/230 ≥ 1.0, RIN ≥ 6.5, 28S:18S ratio ≥ 1.0, and total RNA amount > 10 µg. Gene expression differences and GO/KEGG functional enrichment were performed on the Majorbio I‐Sanger Cloud Platform (www.i‐sanger.com), applying the Benjamini‐Hochberg method for *P*‐value correction. The original sequencing data has been uploaded to NCBI's SRA database for storage (SRA: PRJNA1181585).

### Cell Culture and Stimulation

RAW264.7 cells were seeded in 24‐well plates with a density of 2 × 10⁵ cells per well and cultured in Dulbecco's Modified Eagle's Medium F12 (DMEM) supplemented with 10% foetal bovine serum and 1% penicillin‐streptomycin at 37 °C in a 5% CO₂ atmosphere. Groups of baicalin dose screening tests include: CON, LPS (100 ng mL^−1^), BL + LPS (100 ng mL^−1^ LPS and 10, 20, 50, 100, 200, 300 µm baicalin, respectively). LPS and baicalin were added at the same time. After 12 h of treatment, the culture medium was removed and replaced with a new culture medium. 10 µL of CCK‐8 (Solarbio, CA1210) was added to each well in 96‐well plates for 2 h, and the absorbance value was detected to calculate the cell viability. Groups of baicalin supplementation tests include: CON, LPS (100 ng mL^−1^), BL + LPS (100 ng mL^−1^ LPS and 200 µm baicalin). Groups of *Lactobacillus amylovorus* supplementation tests include: CON, LPS (100 ng mL^−1^), *L. am* (100 ng mL^−1^ LPS and 8×10^6^ CFU mL^−1^ *Lactobacillus amylovorus*), Inactivated *L.am* (100 ng mL^−1^ LPS and 8×10^6^ CFU mL^−1^ inactivated *Lactobacillus amylovorus*), Supernate (100 ng mL^−1^ LPS and 200 µL supernate), Chrysin (100 ng mL^−1^ LPS and 20 µm chrysin).

### 16S rRNA Gene Sequencing Analysis

Microbial DNA was extracted from the colon mucosa using the E.Z.N.A. soil DNA Kit (Omega Biotek, Norcross, GA, U.S.) following the manufacturer's instructions. The V3‐V4 regions of the bacterial 16S rRNA gene were then amplified with primers 338F (5′‐ACTCCTACGGGAGGCAGCAG‐3′) and 806R (5′‐GGACTACHVGGGTWTCTAAT‐3′) on an ABI Gene Amp 9700 PCR thermocycler (ABI, CA, USA). The amplicons were sequenced using the Illumina MiSeq platform equipped with PE300 technology. The sequencing data, including chimeric sequence removal, was processed on the Majorbio I‐Sanger Cloud Platform (www.i‐sanger.com, Majorbio, Shanghai, China). This platform used the default parameters to perform further analyses, including alpha‐diversity, unweighted principal coordinate analysis, and beta‐diversity. The original sequencing data has been uploaded to NCBI's SRA database for storage (SRA: PRJNA1181296).

### Isolation and Culture of *Lactobacillus amylovorus* SKLAN202401ZF

Fresh colonic contents samples from piglets in the BL + *E. coli* group were collected to isolate *Lactobacillus amylovorus*. An appropriate volume of colon contents was preserved in glycerol, mixed with nine times the volume of physiological saline, shaken thoroughly to separate the microbiota, and filtered to remove impurities. The filtrate was then serially diluted and plated on MRS agar. After incubation at 37 °C for 48 h, single colonies were selected and striated on MRS agar for purification. The purified colonies were sequenced using universal primers 27F (5′‐AGAGTTTGATCCTGGCTCAG‐3′) 1492R GGTTACCTTGTTACGACTT‐3′). The 16S rRNA gene sequence was compared with the NCBI nucleotide sequence database to identify *Lactobacillus amylovorus*. The 16S RNA sequence of *Lactobacillus amylovorus* SKLAN202401ZF has been submitted to the NCBI GenBank (Accession Number: pq555640). The strain has been preserved at the Institute of Microbiology, Chinese Academy of Sciences (CGMCC NO.30022).

### *Lactobacillus amylovorus* SKLAN202401ZF and Chrysin Supplementation Experiment

Weight‐matched ICR mice (three‐week‐old) were randomly assigned to four groups with 12 mice of each: CON, *E. coli* SKLAN202302 group (*E. coli*), *L. am* + *E. coli*, and chrysin + *E. coli* group. Mice in the *L. am* + *E. coli* group received a daily gavage of 0.2 mL of *Lactobacillus amylovorus* SKLAN202401ZF (concentration: 8×10^6^ CFU mL^−1^) for three weeks, while mice in CON and *E. coli* groups received the same volume of saline. Mice in the chrysin + *E. coli* group received a daily gavage of 10 mg kg^−1^ chrysin. Subsequently, mice in the *L. am* + *E. coli*, chrysin + *E. coli*, and *E. coli* groups were administered with intraperitoneal injections of *E. coli* SKLAN202302 (concentration: 2 × 10^9^ CFU mL^−1^): 0.1 mL initially, followed by 0.2 and 0.3 mL after 24 and 48 h, respectively. The final injection was followed by euthanasia 24 h later to collect colon samples for further analysis, while the control group received saline injections. During the experiment, the mice had unlimited access to food and water.

### Metabolomics Analysis

Non‐targeted metabolomics analysis was performed on the colonic chyme of piglets and mice, *Lactobacillus amylovorus*, culture supernatants, and blank MRS culture medium. The analysis was conducted using a Thermo UHPLC‐Q Exactive HF‐X system, equipped with an ACQUITY HSS T3 column (Waters Corporation, Milford, USA) at Majorbio Bio‐Pharm Technology Co. Ltd. (Shanghai, China). Initial data processing was carried out using Progenesis QI software (Waters Corporation, Milford, USA). The metabolites were identified by searching databases, including HMDB (http://www.hmdb.ca/), Metlin (https://metlin.scripps.edu/), and the Majorbio database. Data analysis was performed using the Majorbio cloud platform (https://cloud.majorbio.com). Metabolites with VIP values greater than 1 and *p*‐values less than 0.05 were identified as significantly different metabolites. VIP scores were determined from the results of the OPLS‐DA model.

### Quantitative Real‐Time Polymerase Chain Reaction (qRT‐PCR) and Western Blotting

Total RNA was extracted from the colonic mucosa as described in transcriptomics. The RNA was quantified using a NanoDrop 2000 spectrophotometer (NanoDrop Technologies, Wilmington, DE, USA) and diluted to a suitable concentration. Next, the PrimeScript RT Reagent Kit (Takara, Shiga, Japan) was used to reverse transcribe RNA into cDNA. Gene expression levels were measured by qRT‐PCR using SYBR Premix Ex Taq (Takara), with *β*‐actin as the internal normalization control. Primer sequences for these analyses are provided in Tables S3 and S4 (Supporting Information).

The total protein from the colonic mucosa was extracted using RIRA buffer (Solarbio, Beijing, China) and was quantified using the bicinchoninic acid protein assay kit (Thermo Fisher Scientific, MA, USA). Protein signals were detected with an enhanced chemiluminescence (ECL) kit (Bio‐Rad, CA, USA), and images were acquired using the Bio‐Rad ChemiDoc XRS imaging system (Bio‐Rad). The primary antibodies used were obtained from the following suppliers: anti‐ACTB (Sangon Biotech, #D110001), NLRP3 (Beyotime, #AF2155), IL‐1β (Bioss, #bs‐25615R), Occludin (Sangon Biotech, #D110001), CD163 (Sangon Biotech, #D260965), STAT2 (Proteintech, #16674‐1‐AP), TLR4 (Beyotime, #AF8187), Foxp3 (Beyotime, #AF6927), RORγt (Bioss, #bs‐10647R), IL‐17F (Bioss, #bs‐7333R), CTLA4 (ABclonal, #A13966), IRF4 (Proteintech, #66451‐1‐Ig), IRF7 (Proteintech, #22392‐1‐AP), and TLR9 (Beyotime, #AF8193).

### Immunofluorescence Analysis

Following the cell treatment on coverslips, the culture medium was removed, and the cells were washed with phosphate‐buffered saline (PBS). The cells were then fixed in 4% paraformaldehyde over 30 min. For surface antibody staining, blocking was performed using PBS buffer containing 5% goat serum and 0.5% Tween‐20 for 30 min before incubating with the primary antibody overnight. The cells were washed with PBS the following day and incubated with the secondary antibody for 1 h. Nuclei were stained with Hoechst for 5 min. Finally, the coverslips were mounted using an anti‐fade reagent. For intracellular proteins, the cells were initially treated with PBS containing 0.5% Triton X‐100 to permeabilize them, followed by blocking, antibody incubation, and staining procedures as described for surface proteins. The primary antibodies used were obtained from the following suppliers: CD86 (Sangon Biotech, #D224476), CD163 (Sangon Biotech, #D260965), IL‐1β (Bioss, #bs‐25615R), TLR4 (Beyotime, #AF8187), NLRP3 (Beyotime, #AF2155), and IRF7 (Proteintech, #22392‐1‐AP). Secondary antibodies for immunofluorescence staining were Cy3‐labeled Goat Anti‐Rabbit IgG (H+L) (Beyotime, #A0516).

### FISH Analysis

For FISH analysis, colon sections were first deparaffinized and rehydrated, then hybridized with Cy3‐labeled probes (5′‐cy3‐GCTTTGGGCATTGCAGACTCCCATGGTGTGACGG‐3′, Sangon Biotech) to analyze *Lactobacillus amylovorus* abundance.

### *Lactobacillus amylovorus* SKLAN202301ZF Whole Genome Sequencing

Genomic DNA was extracted from *Lactobacillus amylovorus* SKLAN202301ZF using a Bacterial DNA extraction kit. Next, sequencing was performed using the PacBio Sequel IIe and Illumina platforms. For Illumina sequencing, DNA was fragmented into 400–500 bp fragments using the Covaris M220. Libraries were constructed using the NEXTFLEX Rapid DNA‐Seq kit and paired‐end sequencing (2 × 150 bp). For PacBio sequencing, DNA was fragmented to ≈10 kb, ligated with SMRTbell adapters, and sequenced using standard protocols. Genome assembly was performed using Unicycler and Pilon, with quality control applied to both Illumina and PacBio reads. Gene prediction and annotation were performed using Glimmer, Prodigal, and GeneMarkS. Functional analysis was conducted using the KEGG database. The original sequencing data has been uploaded to NCBI's SRA database for storage (SRA: PRJNA1228157).

### Statistical Analysis

Statistical analyses were performed using SAS software (version 9.4; SAS Institute, Cary, NC, USA). Data are presented as mean ± standard error of the mean. A one‐way ANOVA was performed to compare differences across the groups, and subsequent multiple comparisons were conducted using the least significant difference method. A significance level was set at ^*^ *p* < 0.05.

## Conflict of Interest

The authors declare no conflict of interest.

## Author Contributions

S.F.Z. and R.Q.Z. contributed equally to this work. S.F.Z., R.Q.Z., L.C., Q.G.M., and H.F.Z. performed conceptualization. S.F.Z., M.Z., K.L., H.X.W., Y.X., D.D.L., R.Q.Z., and L.C. performed methodology. S.F.Z., R.Q.Z., L.C., and H.F.Z performed investigation. S.F.Z., M.Z., K.L., H.X.W., Y.X., and D.D.L. performed visualization. R.Q.Z., L.C., Q.G.M., H.F.Z. performed supervision. S.F.Z. wrote the original draft. S.F.Z., R.Q.Z., L.C., Q.G.M., and H.F.Z. performed wrote, reviewed, and edited the final manuscript.

## Supporting information



Supporting Information

## Data Availability

The raw sequence data generated during this study have been deposited in the NCBI Sequence Read Archive (SRA) under the Bioprojects PRJNA1181585 and PRJNA1181296. All other relevant data supporting the findings of this study are available from the corresponding author upon reasonable request.
