# Supplementary material for: Mechanisms of Baicalin Alleviates Intestinal Inflammation: Role of M1 Macrophage Polarization and Lactobacillus amylovorus
Source: Adv Sci (Weinh). 2025 Apr 8;12(21):2415948. doi: 10.1002/advs.202415948 (PMC12140389; doi:10.1002/advs.202415948)
Supplement: Supplementary file 1 — Supporting Information [file ADVS-12-2415948-s001.docx]

Supplementary Materials for

**Mechanisms of baicalin alleviates intestinal inflammation: Role of M1 macrophage polarization and *Lactobacillus amylovorus***

Shunfen Zhang *et al*.

Corresponding author. Email: chenliang01@caas.cn

**Supplementary Text**

**Table S1 Epithelial damage scoring**

| Score | Basis |
| --- | --- |
| 0 | no epithelial changes observed |
| 1 | presence of small, multifocal superficial epithelial lesions, characterized by vacuolation, signs of apoptosis, and decay/necrosis at the tips of the villi |
| 2 | moderate, multifocal superficial epithelial injury exhibiting vacuolation, apoptotic signs, and decay/necrosis of villi tips |
| 3 | severe multifocal epithelial injury, including pseudomembrane formation (characterized by luminal neutrophils and exfoliated epithelium in a fibrin matrix) |
| 4 | equivalent to score 3 but with a distinctive pseudomembrane or epithelial ulceration indicating complete loss of focal epithelium |

**Table S2 Inflammation scoring**

| Score | Basis |
| --- | --- |
| 0 | no inflammation present |
| 1 | small, multifocal neutrophilic inflammation without clustering, presenting as scattered cells |
| 2 | moderate, multifocal neutrophilic inflammation with more pronounced submucosal involvement |
| 3 | severe, multifocal to coalescing neutrophilic inflammation, with extensive submucosal and intestinal wall involvement |
| 4 | similar to score 3 but includes an abscess or extensive involvement of the intestinal wall |

**Table S3 List for PCR primers of piglets**

| Gene number | Gene name | Primer (5^,^→3^,^) | |
| --- | --- | --- | --- |
| NM_213948.1 | *IFN-γ* | F: GCTCTGGGAAACTGAATGACTTCG  R: CTGACTTCTCTTCCGCTTTCTTAGG |  |
| NM_214041.1 | *IL-10* | F: CCGACTCAACGAAGAAGG  R: CTGGTTGGGAAGTGGATG |  |
| NM_001113039.2 | *TLR4* | F: GCCTCCAAACCTTGAAAA  R: GAATGAAATGCCCTCTGG |  |
| NM_213958.1 | *TLR9* | F: TGCTTCCACCTGTGCCT  R: ACCCGCAGCTCGTTGTA |  |
| NM_213835.1 | *CD25* | F: TCCTCAGCATCCTCCTCCTCAG  R: AGCATCCAGCCCGCATACAG |  |
| NM_001128438.1 | *FOXP3* | F: ACACCCAGGAAAGACAGCA  R: TCTCACAACCAGGCCACTT |  |
| NM_011281.3 | *RORγt* | F: CTGATGGGCTGTCCGAGT  R: GGCAGAGACCACCTTCCA |  |
| XM_013978026.2 | *IL-17F* | F: AGGCAACACAGGCACAGAGG  R: CATCAACAGCAGCAGGGACTTG |  |
| NM_214022.1 | *TNF-α* | F: CCGACAGATGGGCTGTA  R: TCTTGATGGCAGAGAGGAG |  |
| NM_214055.1 | *IL-1β* | F: CCAGCCAGTCTTCATTGTT  R: CATCTCTTTGGGGCCAT |  |
| XM_021086047.1 | *ACTB* | F: CATCGTCCACCGCAAAT  R: GCCATGCCAATCTCATCTC |  |

**Table S4 List for PCR primers of mice**

| Gene number | Gene name | Primer (5^,^→3^,^) |
| --- | --- | --- |
| NM_009283.4 | *STAT1* | F: CAGTGCTGCCCCATAATAC  R: CTCTGCGTCCCTGTTTTC |
| NM_019963.1 | *STAT2* | F: GACCCATTCCGCTGTTC  R: GCGCCATTTGGACTCTT |
| NM_213660.3 | *STAT3* | F: GTTGGAGCAGCATCTTCAG  R: TGCATCTTCTGTCTGGTCA |
| NM_011905.3 | *TLR2* | F: TCCAGGCCAAGAGGAAG  R: ATGAGGTTCTCCACCCAAT |
| NM_126166.4 | *TLR3* | F: GGGATTGGTGAGTCTGAAGT  R: AGTGAGCAAGGGAGAATGAG |
| NM_013674.1 | *IRF4* | F: TCTAAGCCCGACAGGAAA  R: GCCTATCCATCCAACGC |
| NM_001252601.1 | *IRF7* | F: GCGAAGAGAGCGAAGAGG  R: GGCCCACAGTAGATCCAAG |
| NM_008320.4 | *IRF8* | F: GGTGGTCCTGTGCTTCG  R: CTCTTGCCCGCTTCCTC |
| NM_001159417.1 | *IRF9* | F: CCCTCAACAAGAGTTCCG  R: GGTTCCGTGGTTGGTTAG |
| NM_001360536.1 | *IL-1β* | F: AGTTGACGGACCCCAAA  R: TCTTGTTGATGTGCTGCTG |
| NM_019663.3 | *PIAS1* | F: CGTCAGATGAAGAGGAGGA  R: GGATGAGGGAGGTGTTGA |
| NM_146135.2 | *PIAS3* | F: ACTTTCCTGCCTCCTCTCC  R: TCGCCCGACTGTATGGT |
| NM_001199347.2 | *FOXP3* | F: ACACCCAGGAAAGACAGCA  R: TCTCACAACCAGGCCACTT |
| NM_021297.3 | *TLR4* | F: CTTTGCTTCCTTGGTGTTG  R: ATGATTCTCCTCTTCTTCACG |
| XM_006521737.3 | *CD86* | F: TCCATGTCCAAGGCTCA  R: TGTGAAAGACGAATCAGCA |
| NM_019449.2 | *Unc93b1* | F: CCCACGGAAGAGATCGAC  R: AAAGAGCACCTCAAAGCCA |
| NM_001303244.1 | *IL-12* | F: CATCGTTTTGCTGGTGTCT  R: CATCTTCTTCAGGCGTGTC |
| XM_006526463.1 | *CD226* | F: TGAGAGCAGAGAGTTTGGG  R: GAGACAACATCAGGTGCATT |
| NM_021297.3 | *NLRP3* | F: CTTTATCCACTGCCGAGAG  R: AGCTCATCAAAGCCATCC |
| NM_021297.3 | *IL-18* | F: GGAGGGTTTGTGTTCCAG  R: AATACAGGCGAGGTCATCA |
| NM_145834.3 | *IL-17A* | F: GCTCCTCCACACCTGCTAA  R: GGTCCTTCATGCTGTCTCC |
| NM_145856.2 | *IL-17F* | F: TGGGACTTGCCATTCTG  R: TGCTTTGGGGTTCTTCC |
| NM_011332.3 | *CCL17* | F: AGAAGGACCCATGAAGACC  R: TAATCCAGGCAGCACTCTC |
| NM_013599.4 | *MMP9* | F: AGGCTCTCTACTGGGCGTT  R: GAGGAGTCTGGGGTCTGGT |
| NM_021297.3 | *ACTB* | F: CCTCACTGTCCACCTTCC  R: GGGTGTAAAACGCAGCTC |

**Table S5 Different metabolites in BL + *E. coli* group and *E. coli* group**

| Metabolite | BL+*E.coli* | SD | *E.coli* | SD | *P*_value | Regulate |
| --- | --- | --- | --- | --- | --- | --- |
| Chrysin | 4.23 | 0.46 | 3.04 | 0.03 | 8E-06 | up |
| N-Cyclohexylformamide | 4.40 | 0.34 | 3.32 | 0.62 | 0.0012 | up |
| Alanylglutamine | 3.51 | 0.83 | 2.66 | 0.49 | 0.0349 | up |
| PGD2 ethanolamide | 5.33 | 0.40 | 4.51 | 0.57 | 0.007 | up |
| Nicousamide Pyrotinib Maleate | 4.91 | 0.48 | 4.25 | 0.39 | 0.0135 | up |
| N-Acetyl-D-galactosamine | 5.75 | 0.14 | 5.52 | 0.17 | 0.0179 | up |
| Diphenylamine | 4.60 | 0.34 | 4.25 | 0.16 | 0.0276 | up |
| N-Valylphenylalanine | 5.19 | 0.62 | 4.17 | 0.53 | 0.0051 | up |
| Tyrosyl-Valine | 3.62 | 0.95 | 2.40 | 0.92 | 0.0294 | up |
| 2-Methylene-4-oxopentanedioic acid | 4.36 | 0.69 | 3.49 | 0.68 | 0.0323 | up |
| Galactaric acid | 4.82 | 0.26 | 4.15 | 0.37 | 0.0019 | up |
| D-Leucic acid | 6.39 | 0.38 | 5.84 | 0.33 | 0.0129 | up |
| Cholylglutamic acid | 5.19 | 0.43 | 4.67 | 0.25 | 0.0152 | up |
| 2-(Methylamino)benzoic acid | 4.57 | 0.24 | 3.99 | 0.50 | 0.0145 | up |
| Indole-3-Carboxaldehyde | 6.02 | 0.13 | 5.65 | 0.13 | 1E-04 | up |
| 6-Hydroxyhexanoic Acid | 6.47 | 0.33 | 6.04 | 0.25 | 0.0141 | up |
| Deoxycholyllysine | 4.68 | 0.34 | 4.13 | 0.28 | 0.0052 | up |
| 5-Hexyltetrahydro-2-oxo-3-furancarboxylic acid | 5.22 | 0.40 | 4.76 | 0.30 | 0.0275 | up |
| 1-Pyrroline-5-carboxylic acid | 4.25 | 0.35 | 3.85 | 0.17 | 0.0152 | up |
| Orotidylic acid | 4.36 | 0.19 | 3.91 | 0.13 | 0.0002 | up |
| Deoxycholylglutamic acid | 5.55 | 0.22 | 5.22 | 0.23 | 0.0148 | up |
| N-formimidoyl-glutamic acid | 5.02 | 0.19 | 4.71 | 0.27 | 0.0267 | up |
| 9,10-Epoxystearic acid | 6.43 | 0.19 | 6.11 | 0.12 | 0.002 | up |
| 10-(2,3-Dihydroxypropoxy)-10-oxodecanoic acid | 4.74 | 0.30 | 4.20 | 0.49 | 0.0254 | up |
| Ricinoleic acid | 5.68 | 0.20 | 5.33 | 0.29 | 0.0189 | up |
| L-2-Aminobutyric acid | 5.50 | 0.15 | 5.24 | 0.09 | 0.0018 | up |
| (R)-2-Hydroxystearic Acid | 6.95 | 0.10 | 6.60 | 0.13 | 7E-05 | up |
| L-Pipecolic acid | 6.06 | 0.40 | 5.67 | 0.23 | 0.0419 | up |
| 7-Hydroxyefavirenz | 4.78 | 0.35 | 4.30 | 0.45 | 0.0408 | up |
| 17-Hydroxyprogesterone | 4.69 | 0.13 | 4.41 | 0.22 | 0.0102 | up |
| 3 Hydroxycoumarin | 4.21 | 0.13 | 4.00 | 0.07 | 0.0019 | up |
| N-Acetyl-L-aspartic acid | 4.65 | 0.14 | 4.44 | 0.19 | 0.0358 | up |
| Desmethyldeschlorobenzoyl Indomethacin | 5.11 | 0.15 | 4.91 | 0.09 | 0.0078 | up |
| Indole | 5.17 | 0.11 | 4.85 | 0.11 | 0.0001 | up |
| 4-O-(Indole-3-acetyl)-D-glucopyranose | 4.93 | 0.49 | 4.38 | 0.33 | 0.0277 | up |
| Indole-3-acetyl-myo-inositol | 4.96 | 0.54 | 4.37 | 0.37 | 0.0325 | up |

**Table S6 Different metabolites in *L. am* + *E. coli* group and *E. coli* group of the mice**

| Metabolite | *L. am*+*E. coli* | SD | *E. coli* | SD | *P*_value | Regulate |
| --- | --- | --- | --- | --- | --- | --- |
| Chrysin | 4.28 | 1.48 | 1.37 | 0.49 | 0.00102 | up |
| Soyasapogenol A | 1.72 | 1.52 | 0.01 | 0.00 | 0.02012 | up |
| Deoxycholylserine | 2.96 | 0.42 | 1.40 | 1.40 | 0.02598 | up |
| Dihydroxyhexadecanoic Acid | 2.65 | 0.36 | 1.61 | 0.94 | 0.02954 | up |
| Floionolic Acid | 5.20 | 0.14 | 4.86 | 0.33 | 0.04126 | up |
| Val Arg | 2.73 | 0.48 | 2.04 | 0.43 | 0.02658 | up |
| Gly Leu Phe | 2.89 | 0.48 | 2.29 | 0.28 | 0.02335 | up |
| Monoketocholic Acid | 4.14 | 0.19 | 3.62 | 0.36 | 0.01165 | up |
| Dl-Arginine | 4.17 | 0.35 | 3.67 | 0.42 | 0.04869 | up |
| Diflucortolone Valerate | 2.76 | 0.19 | 2.35 | 0.28 | 0.01309 | up |
| Ascorbic Acid | 5.12 | 0.25 | 4.67 | 0.32 | 0.02195 | up |
| N-(1-Deoxy-1-Fructosyl) Leucine | 3.52 | 0.21 | 3.12 | 0.24 | 0.01125 | up |
| Fluocortin Butyl | 4.22 | 0.13 | 3.81 | 0.36 | 0.02348 | up |
| Tranexamic Acid | 2.32 | 0.45 | 1.78 | 0.37 | 0.04788 | up |
| 2-Furoic Acid | 4.15 | 0.17 | 3.86 | 0.26 | 0.04355 | up |
| Deoxycholic Acid | 5.19 | 0.19 | 4.90 | 0.20 | 0.02382 | up |
| Chenodeoxycholic Acid | 4.64 | 0.56 | 3.61 | 0.92 | 0.04243 | up |

**Table S7 Metabolites of the supernatant of *Lactobacillus amylovorus* SKLAN202301ZF culture solution**

| Metabolite | *L. am* | | SD | MRS | | | SD | P_value | | Regulate |
| --- | --- | --- | --- | --- | --- | --- | --- | --- | --- | --- |
| N1,N12-Diacetylspermine | 5.37 | | 0.04 | 0.95 | | | 0.45 | 3.77E-10 | | up |
| N1-Acetylspermidine | 4.91 | | 0.05 | 2.22 | | | 0.09 | 1.86E-14 | | up |
| Spermidine | 4.25 | | 0.06 | 2.85 | | | 0.08 | 1.15E-11 | | up |
| Methoxamine | 3.30 | | 0.05 | 2.27 | | | 0.08 | 2.29E-10 | | up |
| Nonadecanoic Acid | 3.58 | | 0.03 | 0.36 | | | 0.30 | 1.51E-10 | | up |
| D-Pyroglutamic Acid | 4.62 | | 0.05 | 1.34 | | | 0.80 | 1.56E-06 | | up |
| 3-Indolehydracrylic Acid | 4.46 | | 0.03 | 1.36 | | | 0.01 | 1.2E-20 | | up |
| 15,16-Dihydroxyoctadeca-9,12-Dienoic Acid | 3.80 | | 0.14 | 0.82 | | | 0.01 | 1.36E-13 | | up |
| Glycerophosphoric Acid | 5.10 | | 0.82 | 2.27 | | | 0.52 | 3.22E-05 | | up |
| N-Methyl-D-Aspartic Acid | 4.83 | | 0.03 | 2.82 | | | 0.17 | 6.45E-11 | | up |
| 7,8-Dihydropteroic Acid | 2.17 | | 0.12 | 0.17 | | | 0.00 | 2.49E-12 | | up |
| N-Stearoyl Aspartic Acid | 1.65 | | 0.43 | 0.01 | | | 0.00 | 3.16E-06 | | up |
| 3-Methylorsellinic Acid | 4.82 | | 1.19 | 2.89 | | | 0.00 | 0.002572 | | up |
| Cis-15-Octadecenoic Acid | 5.14 | | 0.28 | 3.65 | | | 0.09 | 2.14E-07 | | up |
| Gamma-Linolenic Acid | 4.48 | | 0.15 | 3.04 | | | 0.01 | 3.85E-10 | | up |
| Delta-Tocotrienol | 2.09 | | 0.28 | 0.62 | | | 0.01 | 1.75E-07 | | up |
| 3-Tert-Butyladipic Acid | 4.51 | | 0.04 | 3.14 | | | 0.07 | 1.11E-12 | | up |
| Phaseolic Acid | 2.94 | | 0.62 | 1.35 | | | 0.34 | 0.000268 | | up |
| Beta-Guanidinopropionic Acid | 3.64 | | 0.11 | 2.25 | | | 0.17 | 1.21E-08 | | up |
| Cis-9,10-Epoxystearic Acid | 2.89 | | 0.91 | 1.42 | | | 0.45 | 0.005071 | | up |
| 1-Aminocyclobutane Carboxylic Acid | 5.29 | | 0.05 | 4.19 | | | 0.04 | 1.34E-12 | | up |
| Choldienic Acid | 3.42 | | 0.23 | 2.32 | | | 0.07 | 6.23E-07 | | up |
| Octadecanedioic Acid | 2.08 | | 0.78 | 0.69 | | | 0.53 | 0.004873 | | up |
| Floionolic Acid | 6.24 | | 0.37 | 5.12 | | | 0.03 | 2.01E-05 | | up |
| Methoxyacetic Acid | 6.20 | 0.33 | | | 5.12 | 0.03 | | | 1.23E-05 | up |
| Chenodeoxycholic Acid | 3.30 | 0.62 | | | 0.91 | 0.63 | | | 6.27E-05 | up |
| 7-Ketolithocholic Acid | 2.83 | 1.18 | | | 1.03 | 0.17 | | | 0.004193 | up |
| Taurocholic Acid | 4.88 | 0.02 | | | 3.78 | 0.03 | | | 1.12E-14 | up |
| Deoxycholic Acid | 3.90 | 1.15 | | | 2.39 | 0.10 | | | 0.009437 | up |
| Lactic Acid | 5.27 | 0.05 | | | 4.29 | 0.02 | | | 0.0001 | up |

**Table S8 β-glucuronidase gene in the genome of *Lactobacillus amylovorus* SKLAN202301ZF**

| Gene ID | Location | Family | Family Description | Coverage(%) | Evalue |
| --- | --- | --- | --- | --- | --- |
| gene0234 | Chromosome | GH1 | beta-glucosidase (EC 3.2.1.21); beta-galactosidase (EC 3.2.1.23); beta-mannosidase (EC 3.2.1.25); beta-glucuronidase (EC 3.2.1.31); beta-xylosidase (EC 3.2.1.37); beta-D-fucosidase (EC 3.2.1.38); phlorizin hydrolase (EC 3.2.1.62); exo-beta-1,4-glucanase (EC 3.2.1.74); 6-phospho-beta-galactosidase (EC 3.2.1.85); 6-phospho-beta-glucosidase (EC 3.2.1.86); strictosidine beta-glucosidase (EC 3.2.1.105); lactase (EC 3.2.1.108) | 99 | 1.3E-153 |
| gene0656 | Chromosome | GH1 | beta-glucosidase (EC 3.2.1.21); beta-galactosidase (EC 3.2.1.23); beta-mannosidase (EC 3.2.1.25); beta-glucuronidase (EC 3.2.1.31); beta-xylosidase (EC 3.2.1.37); beta-D-fucosidase (EC 3.2.1.38); phlorizin hydrolase (EC 3.2.1.62); exo-beta-1,4-glucanase (EC 3.2.1.74); 6-phospho-beta-galactosidase (EC 3.2.1.85); 6-phospho-beta-glucosidase (EC 3.2.1.86); strictosidine beta-glucosidase (EC 3.2.1.105); lactase (EC 3.2.1.108) | 99 | 2.5E-133 |
| gene0659 | Chromosome | GH2 | beta-galactosidase (EC 3.2.1.23); beta-mannosidase (EC 3.2.1.25); beta-glucuronidase (EC 3.2.1.31); alpha-L-arabinofuranosidase (EC 3.2.1.55); mannosylglycoprotein endo-beta-mannosidase (EC 3.2.1.152); exo-beta-glucosaminidase (EC 3.2.1.165) | 57 | 2.1E-26 |
| gene0660 | Chromosome | GH2 | beta-galactosidase (EC 3.2.1.23); beta-mannosidase (EC 3.2.1.25); beta-glucuronidase (EC 3.2.1.31); alpha-L-arabinofuranosidase (EC 3.2.1.55); mannosylglycoprotein endo-beta-mannosidase (EC 3.2.1.152); exo-beta-glucosaminidase (EC 3.2.1.165) | 95 | 2.9E-139 |
| gene1389 | Chromosome | GH1 | beta-glucosidase (EC 3.2.1.21); beta-galactosidase (EC 3.2.1.23); beta-mannosidase (EC 3.2.1.25); beta-glucuronidase (EC 3.2.1.31); beta-xylosidase (EC 3.2.1.37); beta-D-fucosidase (EC 3.2.1.38); phlorizin hydrolase (EC 3.2.1.62); exo-beta-1,4-glucanase (EC 3.2.1.74); 6-phospho-beta-galactosidase (EC 3.2.1.85); 6-phospho-beta-glucosidase (EC 3.2.1.86) | 99 | 3.7E-135 |
| gene1392 | Chromosome | GH1 | beta-glucosidase (EC 3.2.1.21); beta-galactosidase (EC 3.2.1.23); beta-mannosidase (EC 3.2.1.25); beta-glucuronidase (EC 3.2.1.31); beta-xylosidase (EC 3.2.1.37); beta-D-fucosidase (EC 3.2.1.38); phlorizin hydrolase (EC 3.2.1.62); exo-beta-1,4-glucanase (EC 3.2.1.74) | 99 | 2.8E-142 |
| gene1508 | Chromosome | GH3 | beta-glucosidase (EC 3.2.1.21); xylan 1,4-beta-xylosidase (EC 3.2.1.37); beta-glucosylceramidase (EC 3.2.1.45); beta-N-acetylhexosaminidase (EC 3.2.1.52); alpha-L-arabinofuranosidase (EC 3.2.1.55); glucan 1,3-beta-glucosidase (EC 3.2.1.58) | 60 | 4.6E-46 |
| gene1568 | Chromosome | GH1 | beta-glucosidase (EC 3.2.1.21); beta-galactosidase (EC 3.2.1.23); beta-mannosidase (EC 3.2.1.25); beta-glucuronidase (EC 3.2.1.31); beta-xylosidase (EC 3.2.1.37); beta-D-fucosidase (EC 3.2.1.38); phlorizin hydrolase (EC 3.2.1.62); exo-beta-1,4-glucanase (EC 3.2.1.74); 6-phospho-beta-galactosidase (EC 3.2.1.85); 6-phospho-beta-glucosidase (EC 3.2.1.86); strictosidine beta-glucosidase (EC 3.2.1.105); lactase (EC 3.2.1.108) | 99 | 5.3E-147 |
| gene1946 | Chromosome | GH1 | beta-glucosidase (EC 3.2.1.21); beta-galactosidase (EC 3.2.1.23); beta-mannosidase (EC 3.2.1.25); beta-glucuronidase (EC 3.2.1.31); beta-xylosidase (EC 3.2.1.37); beta-D-fucosidase (EC 3.2.1.38); phlorizin hydrolase (EC 3.2.1.62); exo-beta-1,4-glucanase (EC 3.2.1.74) | 99 | 4.4E-152 |

**Table S9 Phosphomannomutase and phenolic acid decarboxylase gene in the genome of *Lactobacillus amylovorus* SKLAN202301ZF**

| Gene ID | Gene Name | Gene Description | Location | COG Description | KO Description |
| --- | --- | --- | --- | --- | --- |
| gene1483 | *hxpB* | HAD family phosphatase | Chromosome | Beta-phosphoglucomutase, HAD superfamily | mannitol-1-/sugar-/sorbitol-6-/2-deoxyglucose-6-phosphatase [EC:3.1.3.22 3.1.3.23 3.1.3.50 3.1.3.68] |
| gene1579 | *glmM* | phosphoglucosamine mutase | Chromosome | Phosphomannomutase | phosphoglucosamine mutase [EC:5.4.2.10] |
| gene1613 | *pgm* | phospho-sugar mutase | Chromosome | Phosphomannomutase | phosphoglucomutase [EC:5.4.2.2] |
| gene2092 | *pgmB* | beta-phosphoglucomutase | Chromosome | Beta-phosphoglucomutase, HAD superfamily | beta-phosphoglucomutase [EC:5.4.2.6] |
| gene0526 | *pdc* | phenolic acid decarboxylase | Chromosome | Phenolic acid decarboxylase | phenolic acid decarboxylase [EC:4.1.1.-] |

**Supplementary Figures**


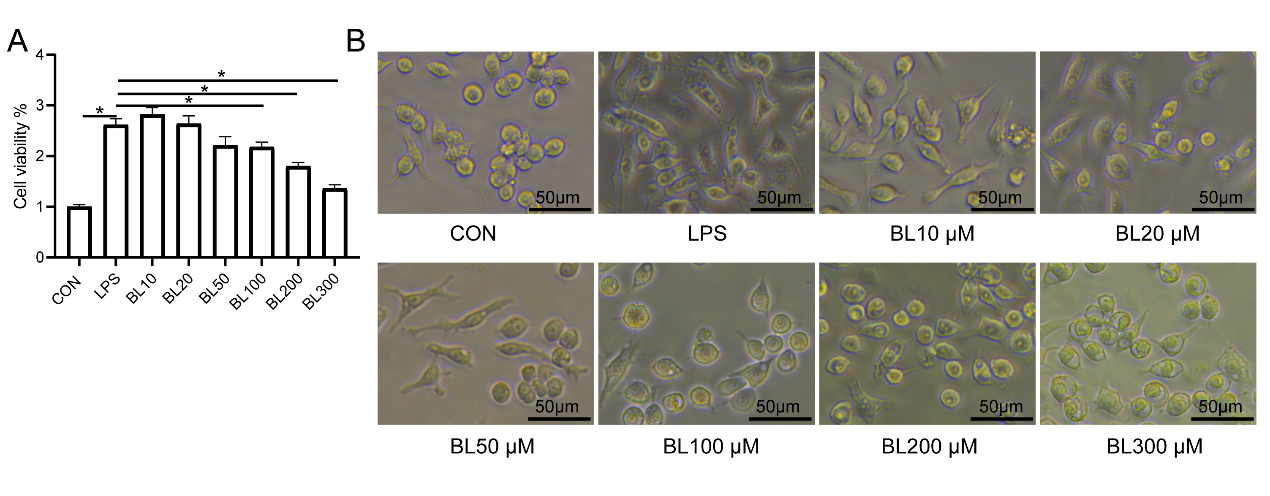


**Figure S1.** Effect of baicalin on macrophage polarization. (**A**) Cell viability of RAW264.7 in CON, LPS (100 ng/mL), BL (10, 20, 50, 100, 200, 300μM) groups. (**B**) Macrophages in CON, LPS, and BL + LPS group.


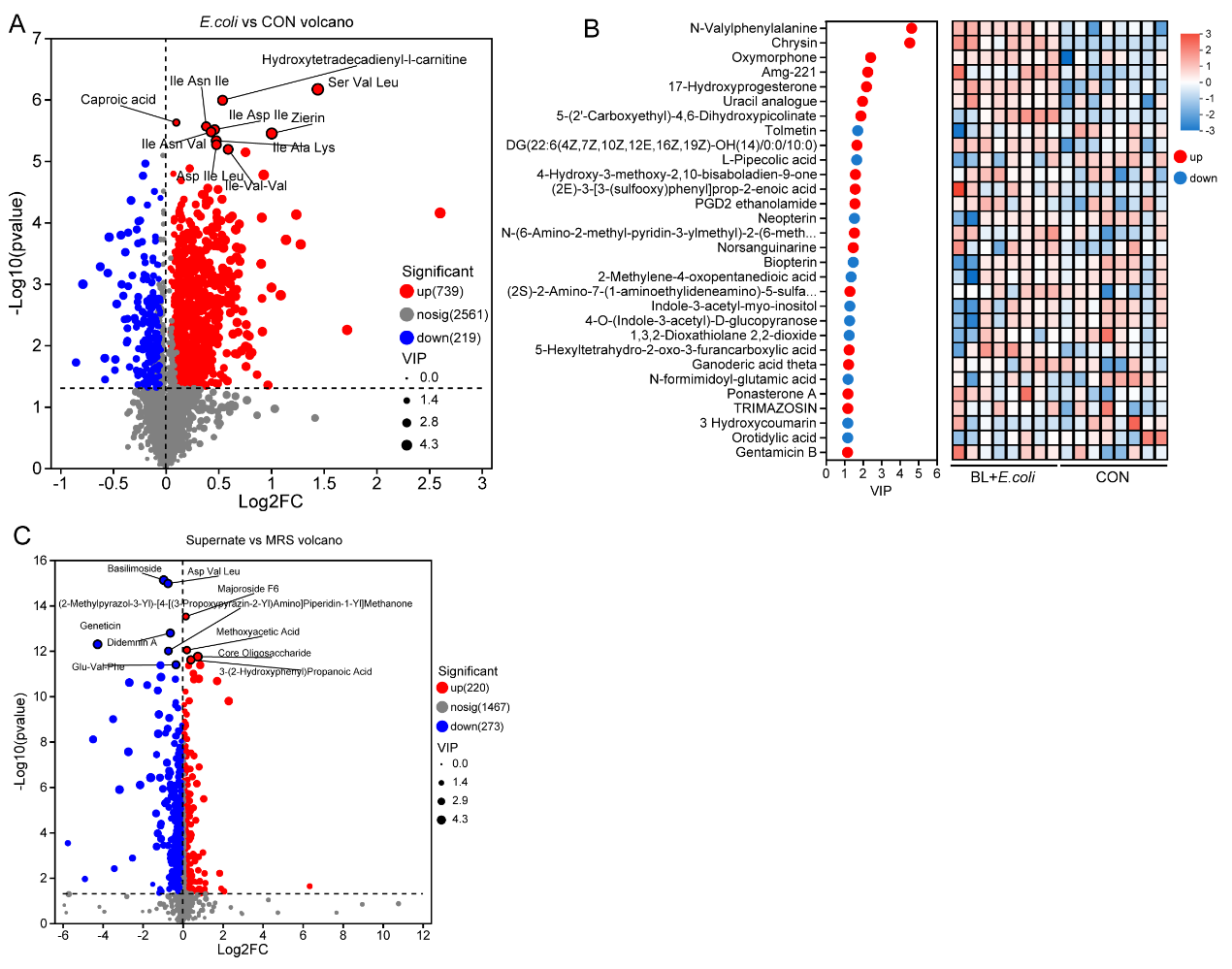


**Figure S2.** Baicalin regulates colonic metabolism disrupted by *E. coli* infection. (**A**) Volcano of differential metabolites in *E. coli* vs CON of the piglets. Each dot represents an individual metabolite. Red dots represent the significantly up-regulated metabolites, blue dots represent the significantly down-regulated metabolites, and gray dots represent non-significant differential metabolites. (**B**) Expression profile and VIP analysis of metabolites for BL + *E. coli* and CON group of the piglets. (**C**) Volcano of differential metabolites in *Lactobacillus amylovorus* SKLAN202301ZF culture and MRS broth medium.
